# Structural insights into the ligand modulation of MRGPRX4 conformational plasticity

**DOI:** 10.1038/s41421-026-00929-x

**Published:** 2026-10-13

**Authors:** Mengna Zhang, Yongxia Wang, Yun Huang, Yaqi Liang, Pengxiao Chen, Zheng Ji, Minghu Song, He Chen, Chunyu Wang, Can Cao

**Affiliations:** 1 Center for Advanced Interdisciplinary Science and Biomedicine of IHM, Division of Life Sciences and Medicine, University of Science and Technology of China, Hefei, Anhui China; 2 Institute of Health and Medicine, Hefei Comprehensive National Science Center, Hefei, Anhui China; 3 Department of Medicinal Chemistry, Tianjin Key Laboratory on Technologies Enabling Development of Clinical Therapeutics and Diagnostics, School of Pharmacy, Tianjin Medical University, Tianjin, China

**Keywords:** Cryoelectron microscopy, Developmental biology

## Abstract

Chronic pruritus is a debilitating condition with limited treatment options, particularly in cholestatic liver diseases, where bile acid dysregulation is a known trigger. Although Mas-related G protein-coupled receptor X4 (MRGPRX4) has recently been identified as a key mediator of bile acid-induced pruritus, the structural mechanisms underlying its inhibition remain poorly understood, hindering the development of therapeutic antagonists. Here, we report the cryo-electron microscopy structures of MRGPRX4 in three distinct states: agonist-bound state, antagonist-bound state, and apo state. Our structures reveal a previously unrecognized antagonist-binding site that is structurally distinct from those engaged by agonists. Complementary functional assays and molecular dynamics simulations further demonstrate that the extracellular pocket of MRGPRX4 undergoes pronounced ligand-dependent conformational rearrangements upon binding to both the agonist and the antagonist. Collectively, these findings elucidate the conformational plasticity underlying MRGPRX4 activity modulation and establish a structural framework for the rational design of MRGPRX4 antagonists that target chronic pruritus.

## Introduction

Mas-related G-protein coupled receptor X (MRGPRX) is a subfamily of class A G-protein coupled receptors (GPCRs) that comprises four members (MRGPRX1–4)^1^. This subfamily was first identified in the millennium^2,3^ but has recently attracted significant attention because of its role in the sensation of itch and pain. Itch is a distressing sensation that can cause discomfort and is a common symptom in many diseases^4–6^. Chronic pruritus, a type of persistent itching that lasts for more than 6 weeks, can severely impair a patient’s quality of life^7,8^. Approximately 22% of the global population is estimated to experience chronic pruritus at some point in their lives^9–13^. Given the multifaceted pathophysiological mechanisms underlying this prevalent condition, the therapeutic management of pruritus can be quite challenging^5^.

Disruption of bile acid metabolism is a hallmark of cholestatic liver diseases^14–16^. Cholestatic pruritus is a specific type of hepatic pruritus associated with dysfunction in bile acid formation, secretion, or excretion and often arises from various underlying etiologies^5^. Recent studies have shown a correlation between bile acid levels and itching^17–21^. Patients who suffer from cholestatic pruritus have elevated plasma bile acid levels. Moreover, both bile acids and bilirubin can activate the MRGPRX4 receptor on dorsal root ganglion (DRG) neurons to induce pruritus^17–19,21,22^. Consequently, MRGPRX4 has emerged as a potential therapeutic target for cholestatic pruritus. In addition to endogenous bile acids, MRGPRX4 can be activated by exogenous agonists, such as obeticholic acid (OCA)^17^, a first-in-class drug targeting the farnesoid X receptor (FXR). Additionally, MRGPRX4 can also be activated by many FDA-approved drugs, mediating their corresponding itching side effects^23^. Most of these drugs contain acidic moieties, such as dexamethasone phosphate (DEX-P), the phosphate ester prodrug of the glucocorticoid dexamethasone. Given that DEX-P shares a steroidal backbone similar to that of bile acids, elucidating the molecular mechanism through which it binds to and activates MRGPRX4 may provide crucial structural insights into the recognition of steroidal pruritogens by this receptor.

MRGPRX4 is a non-canonical GPCR that lacks many of the conserved motifs typically required for class A GPCR activation, including the CWxP, PIF, and DRY motifs^24^. It also lacks a canonical sodium-binding site, which may contribute to its elevated basal activity^25^. This atypical architecture complicates the mechanistic understanding of its activation and inhibition. Previous studies have reported the structures of MRGPRX4 in complex with various agonists^17,23,26^, providing preliminary insights into its activation mechanisms. Most importantly, an antagonist-bound structure has yet to be reported, posing a key obstacle to the development of MRGPRX4-targeted antipruritic drugs.

Here, we present three cryo-electron microscopy (cryo-EM) structures of the MRGPRX4-coupled miniG_sq_ heterotrimer in the agonist-bound state with DEX-P, the antagonist-bound state with compound 54-4 (cmpd54-4), and the apo state. These structures reveal distinct ligand-binding modes for DEX-P and cmpd54-4, accompanied by substantial conformational rearrangements of the extracellular ligand-binding pockets. Mutagenesis studies and complementary molecular dynamics (MD) simulations further validate the binding site of cmpd54-4 and the observed remodeling of the extracellular segments. Collectively, our integrated structural, mutational, and computational studies of MRGPRX4 reveal a distinct antagonist binding mode of cmpd54-4 and delineate the conformational landscape of MRGPRX4 across different functional states, thereby establishing a solid structural foundation for the rational design of therapeutics targeting this receptor.

## Results

### Cryo-EM structures of MRGPRX4–miniG_sq_ complexes in three states

**Fig. 1:**
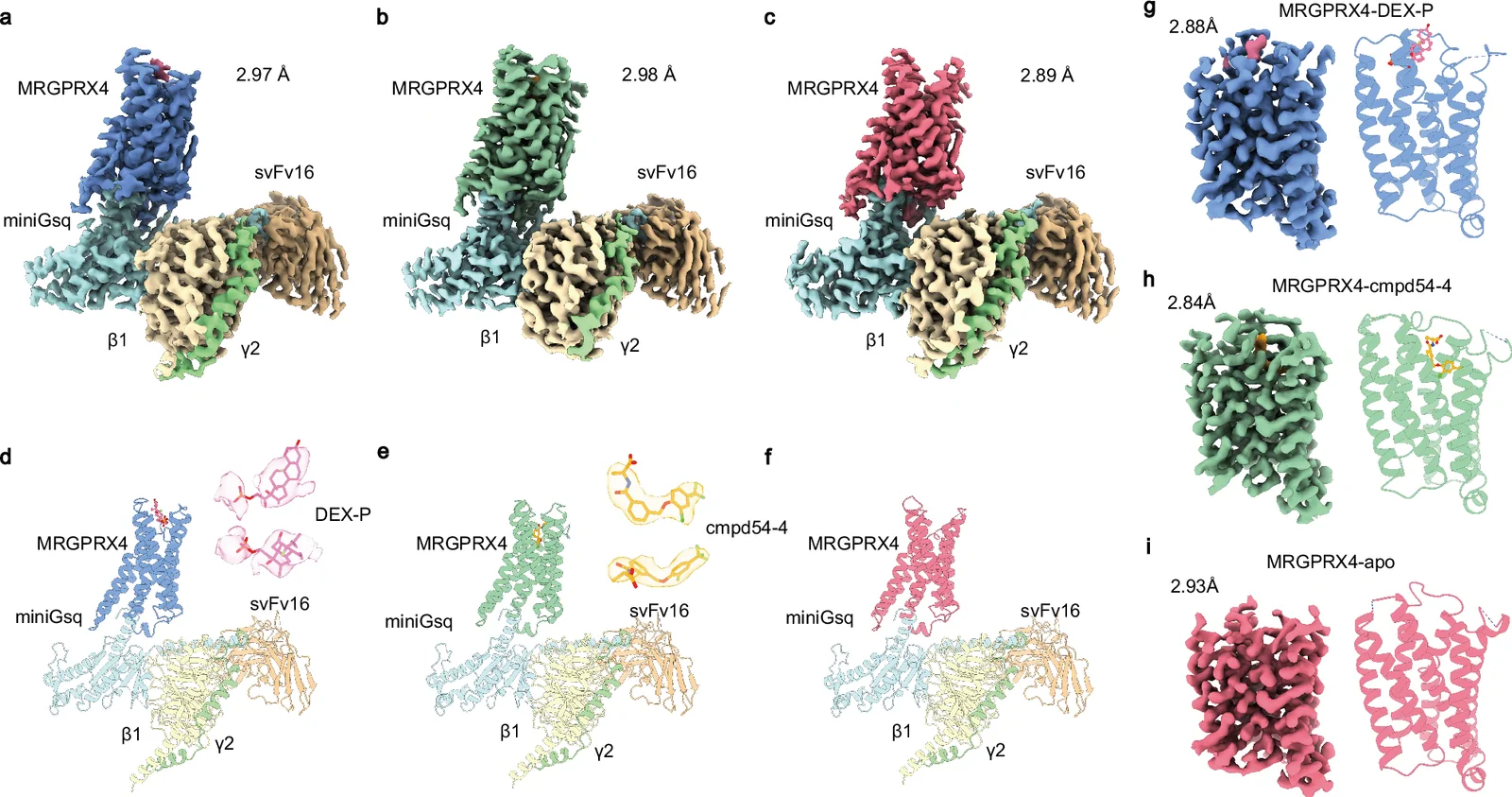
Cryo-EM structures of MRGPRX4 complexes. **a**–**c** Overall cryo-EM density maps of DEX-P–MRGPRX4–miniG_sq_β1γ2–scFv16 (**a**), cmpd54-4–MRGPRX4–miniG_sq_β1γ2–scFv16 (**b**), and apo-MRGPRX4–miniG_sq_β1γ2–scFv16 (**c**). **d**–**f** Cartoon representations of DEX-P–MRGPRX4–miniG_sq_β1γ2–scFv16 (**d**), cmpd54-4–MRGPRX4–miniG_sq_β1γ2–scFv16 (**e**), and apo-MRGPRX4–miniG_sq_β1γ2–scFv16 (**f**). **g**–**i** Local cryo-EM density maps and cartoon representations of DEX-P–MRGPRX4 (**g**), cmpd54-4–MRGPRX4 (**h**), and apo-MRGPRX4 (**i**). DEX-P is presented as pale violet red. Blue, DEX-P-bound MRGPRX4; orange, cmpd54-4; green, cmpd54-4-bound MRGPRX4; pink, apo state MRGPRX4; pale turquoise, miniG_sq_; wheat, Gβ; pale green, Gγ; and burly wood, scFv16. Different ligands and their bound receptors are depicted in contrasting colors to facilitate clear differentiation and visual identification.

We prepared complexes for structural studies using the S83 variant of MRGPRX4 (the most frequent sequence in the National Center for Biotechnology Information (NCBI) database^27^; MRGPRX4 in this text refers specifically to this S83 variant) and an engineered, nucleotide exchange-deficient miniG_sq_ protein (as detailed in “Materials and Methods”). The receptor, miniG_sq_β1γ2 and single-chain variable fragment of mAb16 (scFv16) were co-expressed and assembled into a stable complex even without ligand, a property enabled by its high basal activity^24,28,29^, as well as the use of an engineered miniG_sq_ protein. The stable apo-state complex was incubated with either an agonist (DEX-P) or an antagonist (cmpd54‑4) to generate ligand-bound complexes, enabling us to determine the cryo-EM structures of the MRGPRX4–miniG_sq_β1γ2–scFv16 complex in three states: agonist-bound at 2.97 Å, antagonist-bound at 2.98 Å, and apo at 2.89 Å (Fig. 1a–f; Supplementary Fig. S1 and Table S1). To elucidate the structural details of the receptor and ligand-binding pockets, we performed local refinement focused on the transmembrane region for all three complexes (Fig. 1g–i). High-resolution maps combined with subsequent functional data enabled the construction of models for most of the seven transmembrane (TM) helices and allowed ligands to be positioned (Fig. 1d–f, g–i; Supplementary Fig. S1f, l, r). Unless otherwise stated, all subsequent structural analyses of MRGPRX4 are based on these locally refined models.

### Recognition of the agonist DEX-P in MRGPRX4

**Fig. 2:**
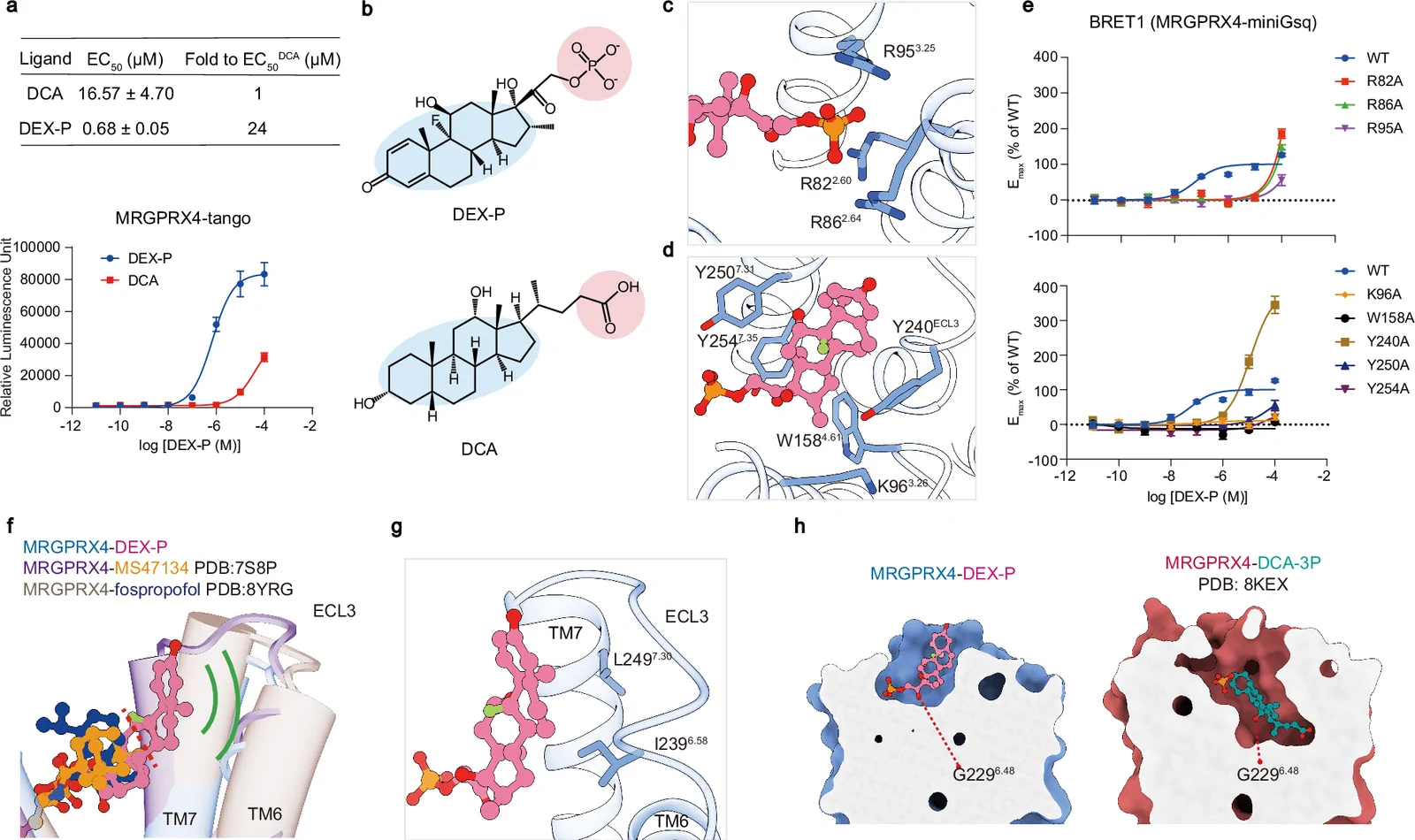
Recognition mechanism of the agonist DEX-P by MRGPRX4. **a** Compared with DCA, DEX-P exhibited increased potency as an MRGPRX4 agonist in the Tango assay. The data are presented as the means ± SEM of three independent experiments performed in duplicate. These data are related to Supplementary Table S2. **b** Chemical structures of DCA and DEX-P. Blue, steroid backbone; light beige, acidic groups. **c**, **d** Binding pocket of DEX-P. Key residues involved in polar interactions with MRGPRX4 (**c**) and in hydrophobic interactions with MRGPRX4 (**d**). **e** Alanine substitution of MRGPRX4 residues that interact with DEX-P reduces miniG_sq_ recruitment. The data are presented as the means ± SEM of three independent experiments performed in duplicate. These data are related to Supplementary Tables S3, S6, S7. **f** DEX-P engages in additional hydrophobic interactions with ECL3 compared with MS47134 and fospropofol. **g** A view from the extracellular side reveals that the ligand DEX-P interacts with ECL3 and the extracellular regions of TM6 and TM7. Several key residues in the extracellular regions of TM6 and TM7 are shown as sticks. **h** Cut-away views of the ligand-binding pockets in the DEX-P-bound (left) and DCA-3P-bound (right) receptor structures. Red dashed lines indicate the distance between the backbone of G229^6.48^ and the respective ligand.

MRGPRX4 can be activated by bile acids and effectively recruits the downstream G_q_ protein, with deoxycholic acid (DCA) resulting in the greatest activation of MRGPRX4^17,18,20^. The EC_50_ of DCA, as measured by the Tango assay (a β‑arrestin recruitment activation reporter assay that converts ligand-induced GPCR activation into quantifiable reporter gene expression)^30^, was 16.6 μM (Fig. 2a; Supplementary Table S2). We initially attempted to resolve the structure of the MRGPRX4–miniG_sq_ complex bound to the endogenous agonist DCA; however, there was no discernible ligand density for DCA. Previously, we identified DEX-P as an agonist of MRGPRX4^23^. It is a phosphate ester prodrug of the glucocorticoid dexamethasone and shares a similar steroid backbone with DCA (Fig. 2b). Both compounds contain a key acidic functional group — a phosphate in DEX-P and a carboxylic acid in DCA — located at the same position on the core scaffold (Fig. 2b). Compared with that in DCA, the phosphate group in DEX-P enhances its water solubility, facilitating experimental handling and reproducibility. Moreover, compared with DCA, DEX-P is 24-fold more potent at MRGPRX4 (Fig. 2a; Supplementary Table S2). Consequently, we turned to the more potent DEX-P and successfully determined the structure of MRGPRX4 in complex with this agonist. In the resulting cryo-EM map, the DEX-P-binding site was visible, although the density was discontinuous between its phosphate and steroid moieties — a feature that was also observed in the previously reported fospropofol-bound structure^23^ (Fig. 1d).

Architecturally, the overall binding mode of DEX-P in the MRGPRX4 structure closely resembled those of the MS47134- and fospropofol-bound MRGPRX4 structures (Supplementary Fig. S2a, b)^23,26^. Specifically, the negatively charged phosphate group of DEX-P strongly interacted with a positively charged cavity formed by residues R82^2.60^, R86^2.64^, and R95^3.25^ (Fig. 2c; Supplementary Fig. S2c). Although the side-chain density for R95^3.25^ is weak, functional mutagenesis supported its critical role: alanine substitution at this position abolished the concentration-response curve to DEX-P, confirming its position within the binding pocket (Fig. 2e; Supplementary Fig. S2d). Notably, similar salt bridge interactions with the three arginine residues were also observed in other agonist-bound MRGPRX4 structures, suggesting that these charge interactions were critical for agonist-induced MRGPRX4 activation (Supplementary Fig. S2c). On the opposite side of the ligand, the steroid core of DEX-P engaged in a hydrophobic pocket formed mainly by residues K96^3.26^, W158^4.61^, Y240^ECL3^, Y250^7.31^, and Y254^7.35^ (Fig. 2d; Supplementary Fig. S2c). Alanine substitution of these MRGPRX4 residues weakened and, in most cases, abolished DEX-P-stimulated activity (Fig. 2e; Supplementary Tables S3, S6, S7), suggesting that these residues were critical for the recognition of DEX-P. In addition, the steroid scaffold of DEX-P extended further toward the extracellular regions, engaging residues I239^6.58^ and L249^7.30^ and those within extracellular loop 3 (ECL3) in hydrophobic contacts (Fig. 2f, g). Notably, the steroid core binding site was solvent accessible, a feature that appears striking for steroid-like ligands (Supplementary Fig. S2f). Despite sharing a similar steroid backbone with the bile acid analog 3-phosphated deoxycholic acid (DCA-3P; Supplementary Fig. S2e), DEX-P adopted a distinct binding pose within the MRGPRX4 pocket (Supplementary Fig. S2f). Although both ligands interacted with the conserved basic pocket through their anionic phosphate groups, their hydrophobic moieties projected in different directions (Fig. 2h; Supplementary Fig. S2f). The DCA-3P hydrophobic region extended deeper into a cavity formed by TM3–TM6 (Supplementary Fig. S2g), whereas the hydrophobic moiety of DEX-P was oriented toward the extracellular sides of TM5 and TM6. These structural observations revealed a notable difference in ligand recognition between these two steroid-backbone MRGPRX4 agonists.

MRGPRXs exhibit largely non-overlapping ligand recognition profiles^27^. Our findings revealed that the residues involved in binding DEX-P were poorly conserved among paralogs (Supplementary Fig. S3a). The equivalent positions in MRGPRX1 and MRGPRX2 showed minimal or no direct involvement in ligand coordination (Supplementary Fig. S3b), underscoring the structural divergence within the subfamily. Furthermore, a comparative analysis of resolved human MRGPR structures highlighted the unique ligand recognition mechanism of MRGPRX4. In MRGPRX4, a triad of arginine residues (R82^2.60^, R86^2.64^, and R95^3.25^) formed a positively charged pocket that selectively engaged acidic moieties on its ligands. In contrast, the binding pockets of related receptors utilize distinct residues: MRGPRX1 employs E157^4.60^ and D177^5.40^; MRGPRX2 employs E164^4.60^ and D184^5.40^; and MRGPRD relies on R103^3.30^ and D179^5.37^ ^26,31–33^. These key residues differ not only in their chemical properties (basic vs acidic) but also in their spatial arrangements within the binding pocket (Supplementary Fig. S3c).

### Distinct binding mode of the antagonist cmpd54-4

**Fig. 3:**
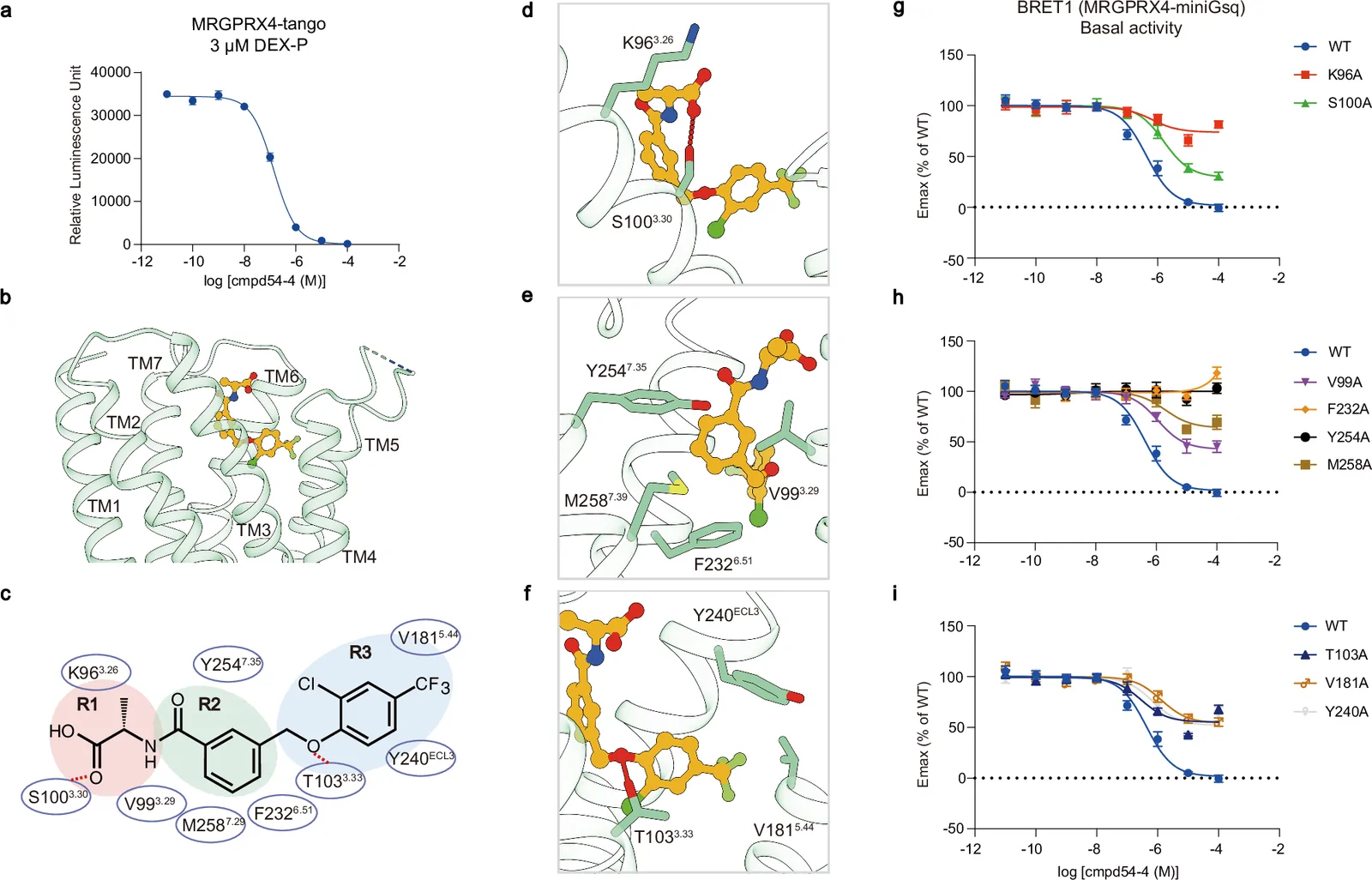
Recognition mechanism of the antagonist cmpd54-4 by MRGPRX4. **a** Cmpd54-4 exhibited antagonist activity against MRGPRX4. A concentration-response curve was constructed for the Tango assay by adding serially diluted cmpd54-4 and 3 μM DEX-P. The data are presented as the means ± SEM of three independent experiments performed in duplicate. These data are related to Supplementary Table S2. **b** Binding site of cmpd54-4. **c** Two-dimensional (2D) interaction diagram of cmpd54-4 in the binding pocket of MRGPRX4. Polar interactions are presented as red dashed lines. The chemical structure of cmpd54-4 was divided into R1 (pink), R2 (green), and R3 (blue). **d**–**f** Residues interacting with regions R1 (**d**), R2 (**e**), and R3 (**f**) of cmpd54-4 are shown. Polar interactions are presented as red dashed lines. **g**–**i** Alanine substitution of MRGPRX4 residues that interact with cmpd54-4 reduced its antagonistic activity. The data are presented as the means ± SEM of three independent experiments performed in duplicate. These data are related to Supplementary Tables S3, S6, S7.

Considering the pivotal role of MRGPRX4 activation in cholestatic pruritus, the development of a selective MRGPRX4 antagonist has substantial therapeutic potential. Since there is no commercially available MRGPRX4 antagonist, we synthesized the antagonist cmpd54-4 on the basis of a patent disclosed by Escient Pharmaceuticals^34^. The antagonist activity of cmpd54-4 was assessed by its ability to block DEX-P-induced activation in the Tango assay (Fig. 3a; Supplementary Table S2). Notably, it also acted as an inverse agonist of MRGPRX4, inhibiting its basal activity in a bioluminescence resonance energy transfer 1 (BRET1) miniG_sq_ recruitment assay (Supplementary Fig. S4a and Table S2). To elucidate the mechanism by which cmpd54-4 inhibited MRGPRX4 activity, we initially attempted to determine the structure of cmpd54-4-bound inactive MRGPRX4. However, despite extensive efforts and the application of multiple structure-stabilization strategies, including the use of an intracellular loop 3 (ICL3)–thermostabilized apocytochrome b562 (BRIL) fusion^35^, the replacement of TM5–TM6 with a κ-opioid receptor (KOR) to facilitate Nb6 binding^36^, and a de novo inactive GPCR design approach^37^, we were unable to obtain a stable MRGPRX4 construct suitable for structural determination.

Inspired by studies on KOR and μ-opioid receptor showing that antagonists can be captured in GPCR–G protein complexes, we applied a similar strategy to MRGPRX4^38,39^. Given that MRGPRX4 is stably pre-coupled with miniG_sq_ even without ligands, we used this pre-formed complex to stabilize and determine the structure of the MRGPRX4–miniG_sq_ complex bound to the antagonist cmpd54-4. While the conformational changes on the intracellular side were constrained by the bound G protein, limiting insights into the full spectrum of inhibitory rearrangements, the structure revealed the extracellular binding mode of cmpd54-4 and provided crucial mechanistic insights into antagonist recognition.

In MRGPRX4, cmpd54-4 adopted an L-shaped conformation within a hydrophobic pocket formed by TM3–TM7 (Figs. 1e, 3b). Notably, its binding site substantially differed from those of agonists (DEX-P, MS47134, and fospropofol) but closely resembled the deep, hydrophobic pocket observed in β_2_AR in complex with the full agonist BI-167107 (Supplementary Fig. S4b). Intriguingly, the binding position of cmpd54-4 showed considerable overlap with that of the agonist DCA-3P, except in the region coordinating the phosphate group, which cmpd54-4 does not occupy (Supplementary Fig. S4c, d). To systematically analyze ligand‒receptor interactions, we divided cmpd54-4 into three regions (R1–R3; Fig. 3c) and analyzed them from structural, functional (assessing the impact on receptor basal activity via alanine substitution), and structure-activity relationship (SAR) perspectives. Region R1 engaged in hydrophobic interactions with the alkyl chain of K96^3.26^, while its carboxyl group formed a polar interaction with S100^3.30^ (Fig. 3d). An alanine substitution of K96^3.26^ nearly abolished the inverse agonistic activity of cmpd54-4, which indicated that K96^3.26^A might impact ligand recognition (Fig. 3g; Supplementary Tables S3, S6, S7). Replacing this methyl group with two bulkier substituents, a benzyl group (cmpd1) or a 4-hydroxybenzyl group (cmp54-7), significantly reduced their inverse agonistic activity (Supplementary Fig. S4f, g). The activity loss likely resulted from these larger substituents forming steric clashes with K96^3.26^, consistent with its functional importance revealed by alanine substitution. In the middle of the ligand, the central R2 segment formed hydrophobic contacts with V99^3.29^, F232^6.51^, Y254^7.35^, and M258^7.39^ (Fig. 3e). Region R3 of cmpd54-4 was enveloped by TM3–TM6 and engaged in extensive hydrophobic interactions with V181^5.44^ and Y240^ECL3^, as well as a hydrogen bond interaction with T103^3.33^ (Fig. 3f). A mutagenesis study revealed that most of these binding pocket residues reduced the inverse agonistic activity of cmpd54-4 (Fig. 3h, i; Supplementary Tables S3, S6, S7). Notably, alanine substitution at F232^6.51^ or Y254^7.35^ completely abolished the inverse agonistic activity of cmpd54-4. To determine whether this loss resulted from impaired antagonist efficacy or from a change in the basal receptor activity of the mutant, we tested the effect of cmpd54-4 on these mutants in the presence of the agonist DEX-P (at EC_80_). Given that the Y254^7.35^A mutation itself abolished the receptor response to DEX-P (Fig. 2e), this assay was performed only on the F232^6.51^A mutant. The results demonstrated that the F232^6.51^A mutation reduced the antagonistic potency of cmpd54-4 but did not abolish it (Supplementary Fig. S4e). To further validate the interaction between the R3 moiety and the receptor, we performed structural modifications. Replacing the R3 group with a phenoxy moiety (cmpd2) or removing it entirely (cmpd3) abolished the inverse agonistic activity (Supplementary Fig. S4f, g and Table S4). A similar loss of activity was observed with cmpd4, a derivative of cmpd54-7 lacking the R3 moiety (Supplementary Fig. S4f, g and Table S4). These results confirmed that hydrophobic interactions mediated by the R3 group with residues from TM3–TM6 were essential for the inverse agonism of cmpd54-4.

Furthermore, we performed structure-based optimization of the inverse agonist cmpd54-4, targeting its R1 and R3 regions — both of which were identified as critical for receptor binding in our prior analysis — to improve their complementarity with the binding pocket and thereby identify analogs with enhanced inhibitory potency (Supplementary Fig. S4f). The alanyl moiety within the R1 region exhibited considerable structural tolerance, as most derivatives, including cmpd54-7, cmpd1, cmpd5, cmpd6, and cmpd7, retained their inverse agonistic activity (Supplementary Fig. S4f–h and Table S4), although none of them showed improved inhibitory potency. We next investigated substitutions on the phenyl group in the R3 region. Analogs bearing a 2,3-dichlorophenyl group (cmpd8) or a 2-amino-4-trifluoromethylphenyl group (cmpd9) displayed weak inhibitory activity (Supplementary Fig. S4f, h and Table S4). Moreover, replacement of the phenyl group with a trifluoromethyl-substituted pyrimidine moiety (cmpd10) resulted in the complete loss of inverse agonistic activity. Although this optimization campaign did not identify inverse agonists with markedly improved potency, the SAR insights and optimization strategies described herein provided a framework for the rational design of future MRGPRX4-targeting antagonists.

### Antagonistic mechanism of cmpd54-4

**Fig. 4:**
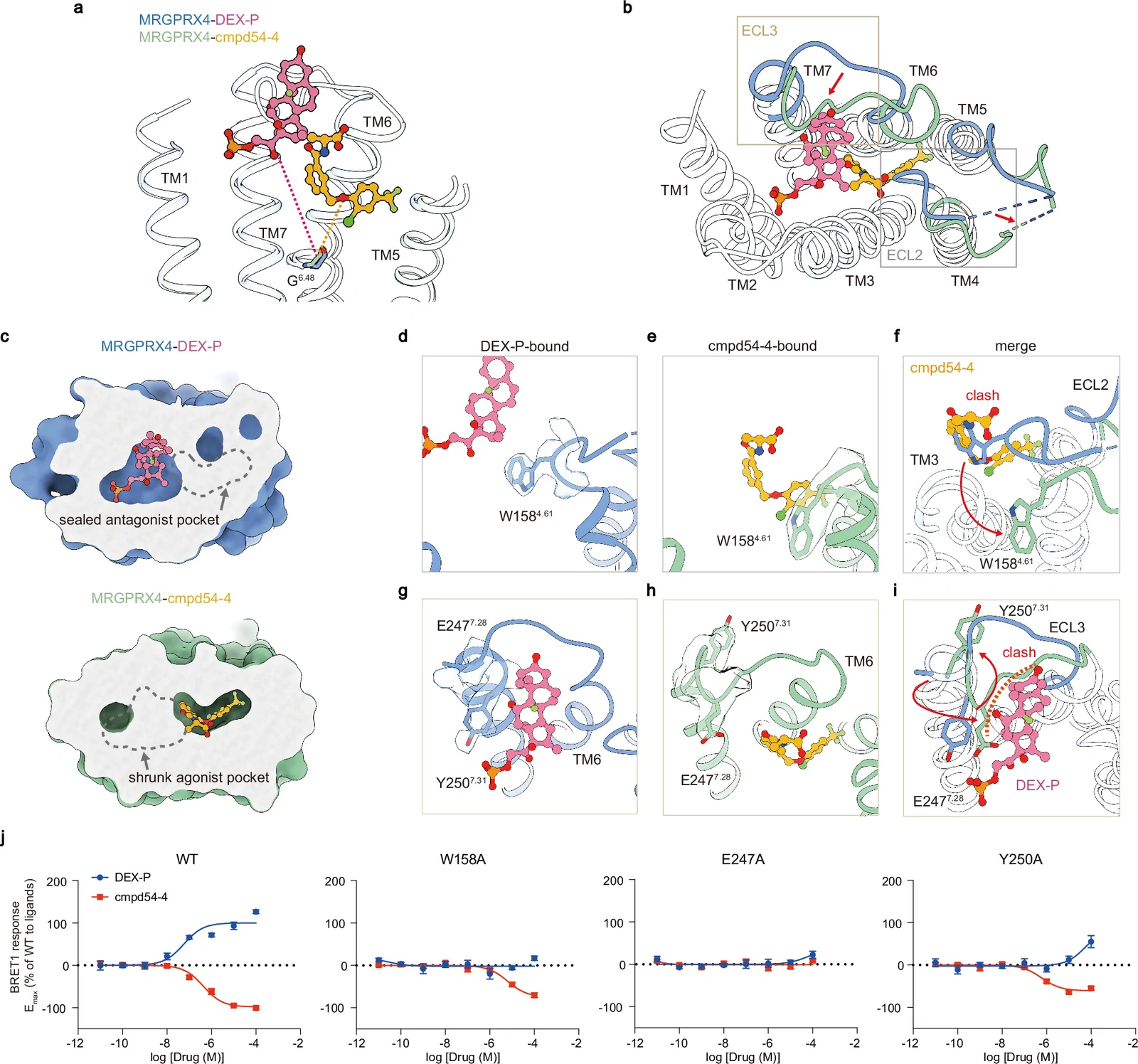
Conformational variations in the extracellular region between DEX-P-bound and cmpd54-4-bound receptor structures. **a** Ligand binding at topographically distinct sites. Red or orange dashed lines indicate the distances between G^6.48^ and the ligands. **b** Superimposed top views of the DEX-P-bound and cmpd54-4-bound MRGPRX4 structures are shown. Red arrows indicate the conformational transition of ECL2 and ECL3 from the DEX-P-bound state to the cmpd54-4-bound state. The regions L238–Y250 and E157–F178 are highlighted as nontransparent cartoons. **c** Cut-away views of the ligand-binding pockets in the DEX-P-bound (left) and cmpd54-4-bound (right) receptor structures. Dark gray dashed contours represent the respective ligand-binding pockets (agonist/antagonist). **d**–**i** Close-up views highlight the structural variations in ECL2 (**d**–**f**) and ECL3 (**g**–**i**). The structures are shown separately for the DEX-P-bound state (**d**, **g**), the cmpd54-4-bound state (**e**, **h**), and as a merged overlay (**f**, **i**). Red arrows indicate the conformational transition of ECL2 and ECL3 from the DEX-P-bound state to the cmpd54-4-bound state. The side chain of E247^7.28^ is not modeled in the DEX-P-bound structure because of a lack of clear density in the map. **j** Functional response curves for DEX-P and cmp54-4 following alanine substitution of key residues. The data are presented as the means ± SEM of three independent experiments performed in duplicate. These data are related to Supplementary Tables S3, S6, S7.

In most class A GPCRs, antagonists and agonists usually occupy the same orthosteric pocket without inducing substantial conformational changes in pocket residues^40^. Antagonists function primarily by directly competing with agonists for the same binding site, thereby preventing agonist engagement. In the case of MRGPRX4, cmpd54-4 binds to the receptor at a spatial position that is structurally distinct from those of known MRGPRX4 agonists, including MS47134, fospropofol, and DEX-P, with little to no overlap between the binding sites (Supplementary Fig. S4b). Moreover, cmpd54-4 stabilizes a receptor conformation that is clearly distinct from that of DEX-P-bound MRGPRX4 (Fig. 4a, b). While DEX-P was situated within a sub-pocket formed by TM1, TM2, TM6, and TM7 and was located far from residue G229^6.48^, cmpd54-4 was bound to another sub-pocket surrounded by TM3–TM6 and was positioned in close proximity to G229^6.48^ (Fig. 4a, b). Notably, this antagonist-binding pocket was inaccessible in the DEX-P-bound MRGPRX4 structure (Fig. 4c), where the ECL2 residue W158^4.61^ projected into the pocket and occluded antagonist binding (Fig. 4d–f). In contrast, in the cmpd54-4-bound structure, W158^4.61^ rotated outward toward the membrane, thereby creating a previously uncharacterized antagonist-specific subpocket that accommodated cmpd54-4 (Fig. 4d–f). This substantial conformational rearrangement of ECL2 resulted in a loss of the direct contact between W158^4.61^ and cmpd54-4 observed in the antagonist-bound state. An alanine substitution of W158^4.61^ reduced the antagonist activity of cmpd54-4 by 1–2 orders of magnitude (Fig. 4j; Supplementary Tables S3, S6, S7). In contrast, the agonist activities of DEX-P, as well as those of fospropofol and MS47134, were completely abolished in the W158^4.61^A mutant, indicating a critical role of W158^4.61^ in agonist-induced receptor activation^23,26^. In addition to W158^4.61^, the residues surrounding this position also underwent significant spatial rearrangements (Supplementary Fig. S5a, b). Surprisingly, a polar interaction network formed above the ligand-binding pocket of cmpd54-4 (Supplementary Fig. S5a). This network was composed of R159^4.62^, which interacted with the backbone of Y240^ECL3^, and Y240^ECL3^, which interacted with the backbones of D177^5.40^ and L156^4.59^ and likely contributed to the stability of ligand binding (Supplementary Fig. S5a). Alanine substitution of these residues resulted in reduced potency and efficacy for both DEX-P and cmpd54-4, underscoring the importance of ECL2-associated interactions in ligand recognition and conformational stabilization of the receptor (Supplementary Fig. S5c and Tables S3, S6, S7).

In addition to ECL2, ECL3 in the cmpd54-4-bound MRGPRX4 structure also underwent substantial conformational rearrangement to shift toward the agonist-binding pocket (Fig. 4b). This movement was accompanied by the uncoiling of the extracellular segment of TM7 and the repositioning of E247^7.28^ into the orthosteric pocket, as observed in agonist-bound structures, together with the outward displacement of Y250^7.31^ toward the membrane (Fig. 4g–i; Supplementary Fig. S5d, e). The side chain of E247^7.28^ was not modeled in the DEX-P-bound structure due to a lack of clear density in the map. Consequently, Y250^7.31^ formed strong interactions with the agonist DEX-P but did not engage with the antagonist cmpd54-4. Consistent with these structural observations, the alanine substitution of Y250^7.31^ abolished the agonist activity of DEX-P, whereas the antagonist activity of cmpd54-4 remained unaffected (Fig. 4j; Supplementary Tables S3, S6, S7). Notably, the carboxylate group of E247^7.28^ functionally mimicked the negatively charged phosphate moieties of MRGPRX4 agonists, including DEX-P, fospropofol, and DCA-3P, thereby facilitating electrostatic interactions with the positively charged cavity formed by R82^2.60^, R86^2.64^, and R95^3.25^ (Supplementary Fig. S5e, f). Consequently, a substantial portion of the agonist-binding pocket was blocked by the inward movement of ECL3, particularly E247^7.28^, which prevented agonist binding (Fig. 4i). Alanine substitution of E247^7.28^ reduced the potency and efficacy of both ligands, suggesting that this residue was important for ligand recognition by both agonists and antagonists (Fig. 4j; Supplementary Tables S3, S6, S7). Consistently, structural superposition with agonist-bound MRGPRX4 revealed severe steric clashes between ECL3 in cmpd54-4-bound receptors and the agonist DEX-P (Fig. 4i). Collectively, these structural observations indicated that binding of the antagonist cmpd54-4 induced substantial conformational rearrangements in the extracellular pocket of MRGPRX4 to sterically prevent agonist binding rather than simply competing for the canonical agonist-binding site.

### Ligand-specific conformational rearrangement in MRGPRX4

**Fig. 5:**
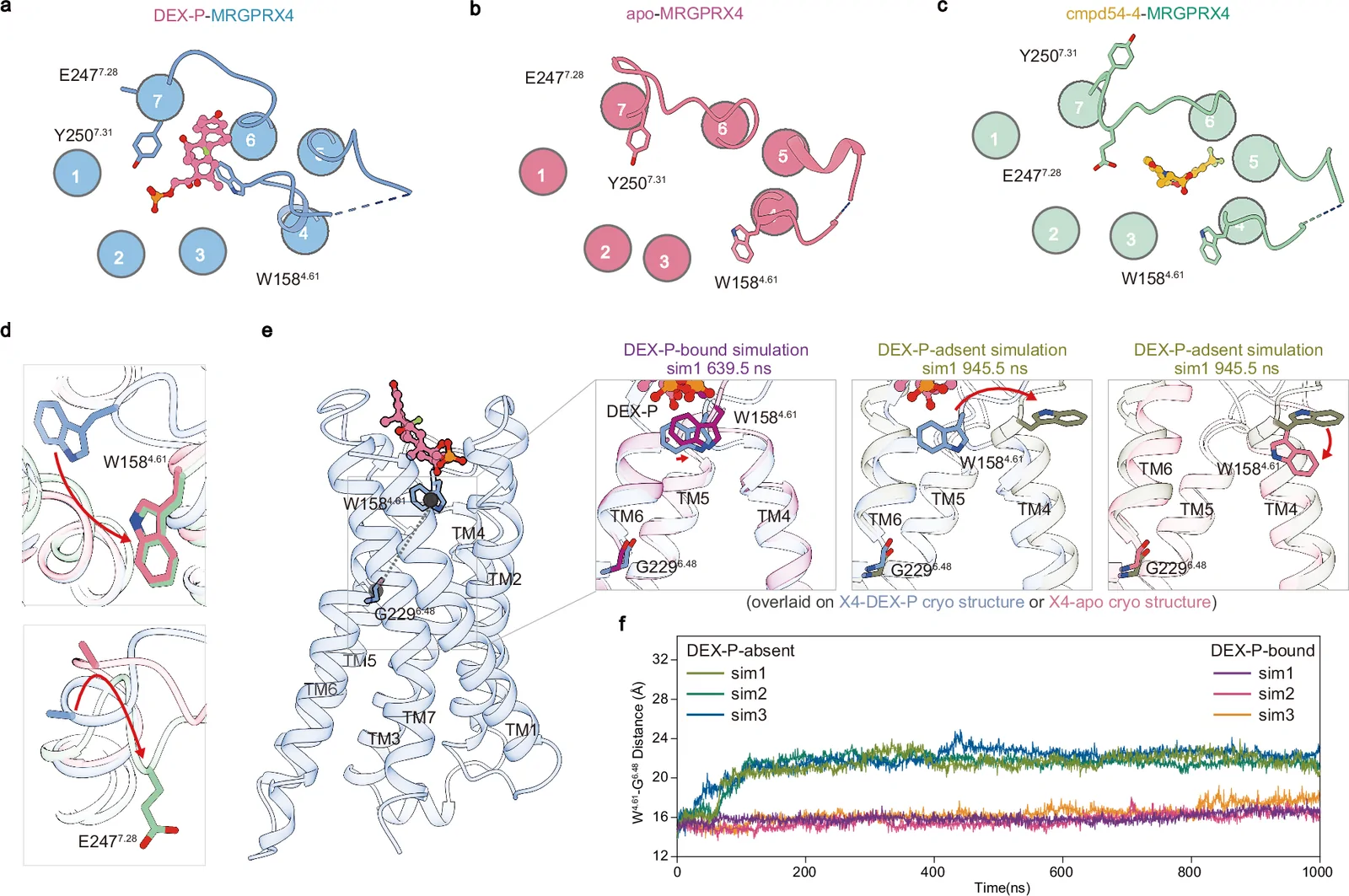
Conformational changes between the three structural states. **a**–**c** Conformational changes in key residues in ECL2 and ECL3 across the DEX-P-bound state (**a**), apo state (**b**), and cmpd54-4-bound state (**c**). **d** Structural overlay showing the repositioning of residues W158^4.61^ (top) and E247^7.28^ (bottom) in the apo, agonist-bound, and antagonist-bound states. Red arrows indicate the direction of the conformational transition from the DEX-P-bound state to the apo state and finally to the cmpd54-4-bound state. **e**–**f** Monitoring W158^4.61^ conformational dynamics. Superposition of representative snapshots from the MD trajectory of W158^4.61^ (colored from cerise to olive drab over time) with the DEX-P-bound (blue) and apo (pink) structures (**e**). The time-dependent distance between the geometric centers of W158^4.61^ and G229^6.48^ is plotted over the course of the simulation for both the DEX-P-absent and DEX-P-bound states (**f**). Each trajectory represents one of three independent simulation repeats.

Given the extensive conformational changes observed in ECL2, particularly at residue W158^4.61^, and in ECL3 at residue E247^7.28^, we sought to determine whether these structural rearrangements were specifically induced by agonist binding or by antagonist engagement. To achieve this goal, we determined the structure of the receptor in its apo state. Notably, from a top-down extracellular view, the position of W158^4.61^ in the apo state resembled that in the antagonist-bound structure, whereas the position of E247^7.28^ more closely matched that in the agonist-bound conformation (Fig. 5a–c). By integrating all three structures, we observed that W158^4.61^ was repositioned from the membrane side to the pocket side by agonist binding, whereas E247^7.28^ was similarly repositioned from the membrane side to the pocket side upon antagonist binding (Fig. 5d).

To gain mechanistic insights into the transition process of W158^4.61^, we performed MD simulations under two conditions: one with the agonist DEX-P bound and another in the absence of ligand, both initiated from the DEX-P-bound conformation (Fig. 5e, f). In the DEX-P-bound simulations, W158^4.61^ displayed minimal positional fluctuations (~1.1 Å) and was stabilized by persistent hydrophobic contacts with the ligand (Fig. 5f). The distance between the geometric centers of W158^4.61^ and G229^6.48^ on TM6 remained stable throughout the simulation trajectory (Fig. 5e). In contrast, in the absence of DEX-P, W158^4.61^ underwent a spontaneous and pronounced conformational transition from an inward-facing orientation to an outward-facing orientation (Fig. 5f). Notably, this outward-facing conformation closely resembled that observed in the experimentally determined apo-state structure. These findings suggested that agonist binding drove W158^4.61^ into the central helical bundle, thereby facilitating hydrophobic contact with DEX-P (Fig. 5e). Furthermore, root mean square fluctuation analysis revealed ECL2 as one of the most dynamically flexible regions across all the systems, providing a molecular basis for the pronounced conformational plasticity associated with W158^4.61^ (Supplementary Fig. S6a–d).

In the apo state, the extracellular region of the MRGPRX4 receptor exhibited significant conformational dynamics, as reflected by poor electron density (Supplementary Fig. S7a). Specifically, residues 86–90 in ECL1 and the side chains of several key residues, such as R86^2.64^, R95^3.25^, R159^4.62^, and Y240^ECL3^, could not be clearly positioned (Supplementary Fig. S7b–d). Consistent with the structural observations, our MD simulations revealed that, compared with ligand-bound systems, the apo system exhibited moderately elevated root mean square deviation (RMSD) fluctuations. The bound ligands DEX-P and cmpd54-4 displayed notably stable behavior, as reflected by their smooth, well-converged RMSD profiles (Supplementary Fig. S6e–j). Together, the results of these analyses indicated that the extracellular region of MRGPRX4 was intrinsically structurally dynamic, enabling the receptor to acquire multiple conformational states and adopt distinct ligand-specific conformations upon binding.

In summary, MRGPRX4 exhibited substantial conformational flexibility in its extracellular region. This structural plasticity allowed the antagonist cmpd54-4 to inhibit receptor activation by inducing a conformational change in residue E247^7.28^. This rearrangement physically blocked the orthosteric binding site, thereby preventing agonist engagement. The observed adaptability of the extracellular domain thereby provided a structural basis for understanding the ligand selectivity and activation mechanisms of MRGPRX4.

### Conformational variations in the G protein coupled to MRGPRX4 across three states

In class A GPCRs, conserved microswitches relay activation signals from the extracellular ligand-binding site to the intracellular G protein-coupling interface, triggering conformational rearrangements that promote nucleotide exchange and G protein activation^41,42^. Despite being coupled to the same engineered miniG_sq_, we observed subtle conformational differences in the receptor and G protein in the antagonist-bound state compared with the apo and active states.

In MRGPRs, residue G/S^6.48^ forms a conserved hydrogen bond with Y^3.36^, which is essential for activation^24^. The distance of this hydrogen bond increases progressively from the DEX-P-bound state to the apo state and then to the cmpd54‑4‑bound state. An alanine substitution at Y106^3.36^A not only altered the baseline response but also abolished ligand binding, confirming its essential role in the activation transition of the receptor (Supplementary Fig. S7e, f). The increased hydrogen bond distance was correlated with a displacement of G229^6.48^, which was accompanied by an outward and downward shift of the CWxP motif in the cmpd54‑4‑bound structure (Supplementary Fig. S7g). This extracellular movement appeared to be poised to induce an inward conformational change on the intracellular side of TM6 but was restrained by the bound G protein. In the PIF motif, the distances among the hydrophobic residues L110^3.40^, L187^5.50^, and F225^6.44^ increased, indicating weakened hydrophobic packing in the antagonist-bound state (Supplementary Fig. S7h). On the intracellular side, although all three structures were coupled to the G_q_ protein, subtle conformational variations were evident. Within the G protein-binding pocket of the three complex structures, side-chain displacements were observed for residues such as R120^3.50^, R131^34.54^, R213^6.32^, and T217^6.36^, revealing a directional trajectory from DEX-P-bound to the apo state and then to the cmpd54‑4‑bound state (indicated by red arrows; Supplementary Fig. S7i). Notably, compared with the other states, the conserved residue R120^3.50^ adopted a distinct side-chain conformation in the cmpd54‑4‑bound structure, suggesting that intracellular conformations could be subtly modulated by extracellular ligand binding even when coupled with the G protein (Supplementary Fig. S7j).

For the G protein of the three complex structures, the α5 helix slightly shifted downward from the DEX-P-bound to apo and then to the cmpd54‑4‑bound state, with residues E355^H5.22^, Y356^H5.23^, and N357^H5.24^ adopting altered side-chain conformations (Supplementary Fig. S7i). Overall, the G protein underwent a swing-like motion that propagated from the α5 helix to the αN helix, with the magnitude of change increasing from minor in the α5 helix to moderate in the h4s6 loop and pronounced in the αN helix (Supplementary Fig. S7k, l).

These observed variations may provide valuable clues about how extracellular ligand binding can allosterically influence the receptor–G protein interface.

## Discussion

MRGPRX4 is a key receptor for bile acids and plays a critical role in cholestatic pruritus. However, the lack of an antagonist-bound structure has hindered the structure-guided discovery of therapeutic MRGPRX4 antagonists. In this study, we report high-resolution cryo-EM structures of human MRGPRX4 in three functional states: bound to the agonist DEX-P, bound to the antagonist cmpd54-4, and in the unliganded (apo) state. Mechanistic insights derived from these structures are rigorously validated through integrated functional assays, SAR studies, and MD simulations.

In previous structural studies, we reported that the conformation of agonist-bound MRGPRX4 diverges significantly from the predicted AlphaFold model. A key distinction is the insertion of ECL2 into the orthosteric pocket upon agonist binding, where it directly interacts with the agonist. Notably, W158^4.61^ is critical for agonist efficacy, as the W158^4.61^A mutation abolishes the response to both the agonist MS47134 and fospropofol^23,26^. Interestingly, in the apo-state structure, W158^4.61^ and ECL2 are positioned outside the binding pocket, indicating that the observed ECL2 conformation in the agonist-bound structure represents an agonist-induced structural rearrangement. In contrast, the antagonist cmpd54-4 exerts its inhibitory effect, in part, by occupying the region where ECL2 binds in the agonist-bound state, thereby preventing the agonist-induced inward movement of ECL2.

Notably, cmpd54-4 does not sterically occlude the agonist-binding site. Instead, it induces a pronounced conformational shift of E247^7.28^, causing it to reorient from the membrane-facing side toward the orthosteric pocket. This movement enables E247^7.28^ to form salt bridge interactions with R82^2.60^, R86^2.64^, and R95^3.25^ within a positively charged subpocket, effectively occluding the phosphate-binding site for DEX-P and further preventing agonist binding (Supplementary Fig. S5d, e). Such extensive antagonist-induced conformational changes are uncommon among class A GPCRs. In aminergic receptors, antagonists and agonists typically occupy overlapping orthosteric sites. Although local helical displacements or side-chain rotamer changes may occur, conformational transitions of this magnitude are rarely observed.

In the NCBI database, L83^2.61^ is annotated as the major variant of MRGPRX4; however, the S83^2.61^ variant is predominant in the human population^27^. We examined the functional impact of this polymorphism by testing the effects of the agonist DEX-P and the antagonist cmpd54-4 on both variants. Our results revealed distinct pharmacological profiles: DEX-P was more potent against the S83^2.61^ variant, whereas cmpd54-4 exhibited similar IC_50_ values against both. This functional observation aligns with our antagonist-bound structure, in which cmpd54-4 binds to a site distal to residue 83 (Supplementary Fig. S5f and Table S5). Therefore, antagonists targeting the cmpd54-4 pocket are likely to be effective regardless of the residue at position 83, suggesting that drug design focused on this binding site may circumvent polymorphism-dependent differences in efficacy.

Compared with those of other MRGPR family members, the agonist-binding site of MRGPRX4 exhibits marked structural divergence from those of MRGPRX1^33,43,44^, MRGPRX2^26,45^, and MRGPRD^31,32^. Intriguingly, the antagonist cmpd54-4 binds at a location that is topologically analogous to the agonist-binding sites in MRGPRX1, MRGPRX2, and MRGPRD. These observations highlight the considerable variability in ligand recognition among MRGPRs.

The limitation of this study is the use of a chimeric, engineered miniG_sq_ protein for both structural and functional assays. Additionally, we used a pre-assembled MRGPRX4–miniG_sq_ complex to determine its structure to capture the ligand binding mode of the inverse agonist cmpd54-4. However, this result might not fully replicate all native interactions between MRGPRX4 and wild-type G_q_. Furthermore, as we were unable to isolate a conformation representing a fully inactive state, the cmpd54-4-bound MRGPRX4–miniG_sq_ complex represents only an intermediate conformation along the activation pathway. This strategy allowed us to elucidate the distinct binding mode of the antagonist, but it limited mechanistic insights into the precise steps of receptor deactivation, as it captured a G protein-engaged intermediate rather than a fully inactive state.

In summary, this study provides a comprehensive structural and mechanistic framework for understanding the pharmacology of MRGPRX4, revealing its unique distinct ligand recognition mode and highly conformational dynamics. Our findings not only provide fundamental insights into the molecular basis of itch signaling mediated by this critical itch receptor but also indispensable structural insights for the rational design of next-generation therapeutics targeting cholestatic pruritus and related conditions.

## Materials and Methods

### Cell lines

The following cell lines were used: *Spodoptera frugiperda* (Sf9, Expression Systems 94-001S) cells in Sf-900 II SFM (Gibco, 10902088) for MRGPRX4 protein expression, human embryonic kidney 293T (HEK293T) cells (ATCC, CRL-11268) for BRET1 recruitment assays, and human Tango luciferase assay (HTLA) cells (a gift from R. Axel) for Tango assays.

### Constructs

The human S83 MRGPRX4 gene sequence was cloned and inserted into the pFastBac1 vector. To increase expression and facilitate purification, a hemagglutinin (HA) signal peptide, a Flag tag, a 10× His tag, a TEV protease cleavage site, and a thermostabilized apocytochrome b562 from *Escherichia coli* (M7W/H102I/R106L, referred to as BRIL) were added at the N-terminus of S83 MRGPRX4. The miniG_sq_ chimera was employed for this study. To overcome the intrinsic instability of Gα_q_, we utilized the stable miniGα_s_^46^ protein as the backbone. Several key residues of the α5 helix were replaced with the corresponding sequence from Gα_q_ to confer G_q_ coupling specificity^47^, while the N-terminal region was substituted with that of Gαi1 to facilitate scFv16 binding. Additionally, the γ2 subunit was attached to the N-terminus of miniG_sq_ using a 3× GSA linker. The resulting miniG_sq_–γ2 and β1 constructs were subsequently cloned and inserted into the pFastBac Dual vector.

### Expression and purification of the MRGPRX4–G protein complex

To express the MRGPRX4 protein complex, bacmids encoding MRGPRX4, miniG_sq_–γ2, β1, and scFv16 were transfected into Sf9 cells using Cellfectin II reagent (Gibco, 10362100). After culturing at 27 °C for 4 days, the P0 baculovirus was harvested. The P0 virus was then used to infect Sf9 cells at a density of 2 × 10^6^ cells/mL and a 1:100 dilution. Following a 3-day incubation at 27 °C with shaking at 120 rpm, the P1 virus was obtained. The supernatant was collected by centrifugation, supplemented with 2% fetal bovine serum (FBS; ExCell Bio, FSP500), and stored at 4 °C in the dark. For protein co-infection, Sf9 cells at a density of 2 × 10^6^ cells/mL were co-infected with baculoviruses encoding MRGPRX4, miniG_sq_β1γ2, and scFv16 at a multiplicity of infection of 2:1:1. The cells were cultured at 27 °C for 48 h, harvested by centrifugation, washed with phosphate-buffered saline (PBS), and stored at –80 °C until use.

The cell pellets were thawed and resuspended in buffer containing 20 mM HEPES (pH, 7.4; Sigma, V900477), 50 mM NaCl (Sigma, 71376), 5 mM MgCl_2_ (Sigma, V900020), 5 mM CaCl_2_ (Sigma, V900269), 100 µM TCEP (Goldbio, TCEP10), 25 mU/mL Apyrase (Sigma, A6132-1KU), and a protease inhibitor cocktail (final concentration: 1 µM E-64 (Macklin, E808962), 0.15 µM aprotinin (Macklin, A800433), and 1 µM leupeptin (Macklin, L812486)), followed by incubation for 1 h at room temperature. The membranes were collected by centrifugation at 25,000 rpm using a Ti45 rotor (Beckman) for 30 min at 4 °C and then solubilized for 4 h at 4 °C in buffer composed of 40 mM HEPES (pH, 7.4), 100 mM NaCl, 10% (w/v) glycerol (Macklin, G810578), 0.5% (w/v) lauryl maltose neopentyl glycol (LMNG; Anatrace, NG310), 0.05% (w/v) cholesteryl hemisuccinate (CHS; Anatrace, CH210), 100 µM TCEP, 25 mU/mL apyrase, and a protease inhibitor cocktail (final concentration: 1 µM E-64, 0.15 µM aprotinin, and 1 µM leupeptin). After centrifugation at 32,000 rpm using a Ti70 rotor (Beckman) for 30 min at 4 °C, the supernatant was collected and incubated overnight with 20 mM imidazole (pH, 8.0; Sigma, V900153) and TALON metal affinity resin (Takara, 635507) at 4 °C with gentle rotation (10 rpm). The resin was loaded onto a gravity column, washed with buffer containing 20 mM HEPES (pH, 7.4), 100 mM NaCl, 5% (w/v) glycerol, 0.01% (w/v) LMNG, 0.001% (w/v) CHS, 100 µM TCEP, and 30 mM imidazole (pH, 8.0), and eluted with the same buffer supplemented with 250 mM imidazole. The eluted protein sample was concentrated to ~500 µL by centrifugation at 800× *g*. Further purification was performed using a Superdex 200 Increase 10/300 column (Cytiva, 28990944) equilibrated with buffer consisting of 20 mM HEPES (pH, 7.4), 100 mM NaCl, 0.00075% (w/v) LMNG, 0.000075% (w/v) CHS, 0.00025% (w/v) glyco-diosgenin (GDN; Anatrace, GDN101), and 100 µM TCEP. The peak fraction was divided and incubated separately with 300 µM DEX-P (Aladdin, 2392-39-4) or 200 µM cmpd54-4 and then concentrated to ~3 mg/mL for cryo‑EM sample preparation.

### Cryo-EM grid preparation and data collection

For cryo-EM sample preparation, 3 μL of the complex was applied to glow-discharged grids (Quantifoil R1.2/1.3, 300 mesh). The grids were blotted for 3.5 s with a blot force of 0 at 4 °C and 100% humidity, followed by rapid vitrification in liquid ethane using a Vitrobot (FEI).

All MRGPRX4–G protein complex datasets were collected on a Titan Krios electron microscope (Thermo Fisher Scientific) operating at 300 kV and equipped with a K3 Summit direct electron detector (Gatan). Micrographs were obtained in counting mode at a pixel size of 0.82 Å and a defocus range of −0.8 μm to −1.6 μm. Each video comprised 40 frames with a total dose of 50 e^−^/Å^2^. Automated single-particle data acquisition was performed using EPU software (Thermo Fisher).

### Cryo-EM data processing

The data were reconstituted in cryoSPARC (v4.4.1)^48^. For the MRGPRX4–cmpd54-4–miniG_sq_ complex, a total of 4404 movies were first subjected to motion correction and contrast transfer function (CTF) patching. Initial sets of 4,865,154 particles, picked by Blob Picker, were extracted with a box size of 88 and then down-sampled by inspection, after which the classes were carefully selected after 2D classification. In addition, a template picker was used on these classes. The group of all the selected particles with duplicates that had already been removed was re-extracted with a box size of 356 and processed for two rounds of ab initio reconstruction, and low-quality particles were excluded from each round. After another three-dimensional (3D) classification with the mask of the receptor moiety, the well-defined classes, including 432,382 particles, were processed for homogeneous and non-uniform refinement. The MRGPRX4–cmpd54-4–miniG_sq_ map was refined to obtain a 2.98 Å resolution map for cmpd54-4-bound MRGPRX4 and the G protein. The cmpd54-4–MRGPRX4 map was refined locally, yielding a 2.84 Å-resolution map for the cmpd54-4-bound receptor. The resolutions were determined using the 0.143 criterion of gold-standard Fourier shell correlation (GSFSC).

Similar processing procedures were used for maps of DEX-P–MRGPRX4–miniG_sq_, DEX-P–MRGPRX4, apo-MRGPRX4–miniG_sq_ and apo-MRGPRX4 with initial sets of 2,300,504 and 1,702,916 particles, respectively. In brief, after several rounds of particle selection and 3D classification with masks, 174,116 and 113,307 particles were selected for the reconstruction of DEX-P-bound and apo-MRGPRX4, respectively. The final refined maps were correspondingly reconstructed to a resolution of 2.97 Å for DEX-P–MRGPRX4–miniG_sq_, 2.88 Å for DEX-P–MRGPRX4, 2.89 Å for apo-MRGPRX4–miniG_sq_, and 2.93 Å for apo-MRGPRX4. The detailed processes are shown in Supplementary Fig. S1b, h, n.

### Model building and refinement

Coordinates for the fospropofol-bound MRGPRX4–miniG_sq_ complex (PDB ID: 8YRG)^23^ were used as initial models that were fit into the cryo-EM density maps using ChimeraX (v1.7)^49^. Real-space refinement was performed via Phenix (v1.21)^50^, and iterative rounds of manual refinement were performed in Coot (v0.9.8)^51^. Validation of the models and cryo-EM maps were also performed with Phenix. The final refinement statistics are provided in Supplemental Table S1. Map/model visualizations and figure preparation were conducted in ChimeraX.

### BRET1 assay

HEK293T cells were cultured in DMEM (Gibco, C11995500BT) supplemented with 10% (v/v) FBS and 100 U/mL penicillin-streptomycin (Gibco, 15140163). In the BRET1 recruitment assay, S83 MRGPRX4 was fused C-terminally to Renilla luciferase (RLuc8) and co-expressed with Venus-tagged miniG_sq_. This miniG_sq_ was engineered on a miniGα_s_ scaffold harboring the α5 helix and αN helix from Gα_q_. Plasmids encoding MRGPRX4–RLuc8 and Venus–miniG_sq_ were co-transfected into HEK293T cells at a ratio of 1:3. At 1 day post-transfection, the cells were seeded into poly-L-lysine (YEASEN, 60716ES08)-coated 96-well white plates (LABSELECT, 11617) in DMEM supplemented with 1% (v/v) dialyzed fetal bovine serum (dFBS; VivaCell, C3820) and cultured overnight. Afterward, the medium was removed, and each well was washed with 60 μL of assay buffer (1× Hank’s balanced salt solution (HBSS), 0.1% (w/v) bovine serum albumin (BSA; BioFroxx, 4240GR100), and 20 mM HEPES (pH, 7.4)). Subsequently, 60 μL of assay buffer containing 5 μM coelenterazine h (Nanolight Technology, 301-1) was added to each well. Following a 5-min incubation, 30 μL of assay buffer containing serially diluted test compounds was added, and the plate was incubated for an additional 15 min in the dark. Luminescence at 480 nm (RLuc8 signal) and fluorescence at 540 nm (Venus signal) were measured using a Tristar 3 Multimode Reader (BERTHOLD). The BRET ratio was calculated as the emission at 540 nm divided by the emission at 480 nm. The data were analyzed using GraphPad Prism software to evaluate the compound-induced activation of MRGPRX4.

### Tango assay

HTLA cells were cultured in DMEM supplemented with 10% (v/v) FBS, 100 U/mL penicillin‑streptomycin, 2 µg/mL puromycin, and 100 µg/mL hygromycin. When the cells reached 60%–70% confluence in 10‑cm dishes, they were transfected with 6 µg of Tango S83 MRGPRX4 plasmid DNA per dish using polyethylenimine (PEI; Polysciences, 24765) at a 1:5 (w/w) DNA:PEI ratio. The DNA‑PEI complex was prepared in 300 µL of Opti‑MEM^TM^ (Gibco, 31985-070), incubated for 1 h at room temperature, and then added to the cells. One day after transfection, the cells were detached, resuspended in DMEM supplemented with 1% (v/v) dFBS and 100 U/mL penicillin‑streptomycin, and seeded into 96‑well plates at 160 µL per well. After 6 h of adhesion, 40 µL of assay mixture containing serially diluted concentrations of the compound and DEX-P at its EC_80_ concentration (3 µM) was added. Following overnight incubation, the medium was aspirated, and 60 µL of Bright‑Glo^TM^ reagent (Promega, E264A), which was diluted 20‑fold in HBSS, was added to each well. The plates were protected from light and incubated at room temperature for 20 min before luminescence was measured using a Tristar 3 Multimode Reader (BERTHOLD).

### ELISA

HEK293T cells were maintained in DMEM supplemented with 10% (v/v) FBS. For transient expression, cells were transfected at ~70% confluency with plasmids encoding Flag-tagged wild-type MRGPRX4, MRGPRX4 point mutants, or an empty pcDNA3.0 vector as a negative control. 24 h post-transfection, the cells were replated into poly-L-lysine-coated 96-well plates at a density of 3–4 × 10^4^ cells per well. The cells were fixed the following day using 4% (w/v) paraformaldehyde (LEAGENE, DF0135) for 10 min at room temperature. After fixation, the wells were washed twice with PBS (120 μL per well) and subsequently blocked with 90 μL of PBS containing 5% (w/v) BSA per well for 1 h at room temperature. The surface expression of the Flag-tagged receptors was detected by incubation with a horseradish peroxidase-conjugated anti-Flag antibody (YEASEN, 30502ES60) diluted 1:10,000 in 5% BSA (60 μL per well) for 1 h at room temperature. Following antibody incubation, the cells were washed three times with PBS (200 μL per wash), and chemiluminescence was initiated by the addition of 80 μL of Super ECL substrate (YEASEN, 36208ES76) per well. After 5 min of incubation, the luminescent signals were quantified using a Tristar 3 Multimode Reader (BERTHOLD). The data were processed and analyzed using GraphPad Prism 10.1 and normalized to signals obtained from wild-type MRGPRX4.

### MD simulations

The initial configurations for MD simulations were derived from high-resolution cryo-EM structures of MRGPRX4 in three distinct states: (A) the DEX-P-bound agonist state, (B) the cmpd54-4-bound antagonist state, and (C) the apo state. Missing amino acids in these structures were completed using the MODELLER program (v10.7)^52^.

All the systems were embedded in a pre-equilibrated palmitoyl-oleoyl phosphatidylcholine (POPC) membrane bilayer generated using CHARMM-GUI^53^. Each system was solvated with explicit TIP3P water molecules, maintaining a minimum water layer thickness of 22.5 Å on both sides of the membrane, and was neutralized with 150 mM NaCl. The AMBER ff14SB and AMBER LIPID 21 parameters^54^ were used for the protein and lipids, respectively. DEX-P and cmpd54-4 were parameterized with the GAFF2 force field^54^, with partial charges assigned using the AM1-BCC scheme and atom types assigned using the Antechamber program^55^. The final systems were constructed as follows: (A) the DEX-P-bound system, comprising a 96 × 96 × 108 Å^3^ box with 18,637 water molecules and 240 POPC molecules; (B) the cmpd54-4-bound system, comprising a 96 × 96 × 106 Å^3^ box with 17,879 water molecules and 240 POPC molecules; (C) the apo system, comprising a 96 × 96 × 108 Å^3^ box with 18,767 water molecules and 240 POPC molecules; and (D) an additional system prepared by removing DEX-P from the DEX-P-bound complex, with identical dimensions and containing 18,665 water molecules and 240 POPC molecules. All the input files from CHARMM-GUI were converted to the GROMACS format using the CHARMM-GUI interface.

MD simulations were performed using GROMACS (v2024.3)^56^ with periodic boundary conditions applied in all directions. Long-range electrostatic interactions were treated using the particle‒mesh Ewald method^57^. The systems were first energy minimized for a maximum of 5000 cycles, with positional restraints applied to protein backbone atoms (4000 kJ/mol/nm^2^), protein side-chain atoms (2000 kJ/mol/nm^2^), membrane lipids (1000 kJ/mol/nm^2^), and ligands when present (1000 kJ/mol/nm^2^). A sequential equilibration protocol was then conducted, starting with 1 ns in the NVT ensemble, followed by 22 ns in the NPT ensemble at 303.15 K (maintained by the V-rescale thermostat^58^) and 1 atm (maintained by the C-rescale barostat^59^). During equilibration, the positional restraints were gradually reduced: the force constants for protein backbone atoms were scaled down from 4000 to 50 kJ/mol/nm^2^, whereas the restraints on protein side chain, lipid, and ligand atoms were removed after 6 ns of the NPT phase. Finally, unrestrained production simulations were carried out for 1000 ns, with trajectory snapshots saved every 500 ps. The processed dataset has been deposited in Zenodo (https://doi.org/10.5281/zenodo.20626130).

### Chemical synthesis of MRGPRX4 compounds

The synthesis and characterization of the compounds are described in Supplementary Methods S1.

## Supplementary information

Supplementary informations-CELLDISC-07915R2

## Data Availability

The coordinates and cryo-EM maps of overall MRGPRX4‒miniG_sq_‒DEX-P, local MRGPRX4‒DEX-P, overall MRGPRX4‒miniG_sq_‒cmpd54-4, local MRGPRX4‒cmpd54-4, overall MRGPRX4‒miniG_sq_-apo and local MRGPRX4-apo have been deposited to Protein Data Bank (PDB) and the Electron Microscopy Data Bank (EMDB) with accession codes PDB: 23CV (EMDB: EMD-68871), PDB: 23CW (EMDB: EMD-68872), PDB: 23CX (EMDB: EMD-68873), PDB: 23CY (EMDB: EMD-68874), PDB: 23CS (EMDB: EMD-68868), and PDB: 23CU (EMDB: EMD-68870), respectively. The previously published structural models used in this study are available from the PDB under the following accession codes: MRGPRX4‒miniG_sq_‒MS47134 complex, 7S8P; MRGPRX4‒miniG_sq_‒fospropofol, 8YRG; MRGPRX4‒DCA-3P complex, 8KEX; β_2_AR‒G_s_‒BI-167107 complex, 3SN6; MRGPRX1‒miniG_sq_‒BAM8-22 complex, 8DWC; MRGPRX1‒miniG_sq_‒compound 16 complex, 8DWH; MRGPRX2‒miniG_sq_‒CST-14 complex, 7S8L; MRGPRX2‒miniG_sq_‒(R)-ZINC-3573 complex, 7S8N; MRGPRD‒G_i_‒β-alanine complex, 7Y12; MRGPRD‒miniG_sq_‒EP-2825 complex, 9DQH. Any additional information required to reanalyze the data reported in this paper is available from the lead contact upon request. Source data are provided with this paper.
