# Supplementary material for: Structural insights into the ligand modulation of MRGPRX4 conformational plasticity
Source: Cell Discov. 2026 Oct 13;12:70. doi: 10.1038/s41421-026-00929-x (PMC13661610; doi:10.1038/s41421-026-00929-x)
Supplement: Supplementary file 1 [file 41421_2026_929_MOESM1_ESM.pdf]

**Supporting Information for**

Structural insights into the ligand modulation of MRGPRX4 conformational plasticity

**This PDF file includes:**

Figures S1 to S7: These supplementary figures provide a structural analysis of the individual apo, agonist-bound, and antagonist-bound states of MRGPRX4, along with a detailed comparison between these states.

Tables S1 to S7: This section comprises the cryo-EM data collection statistics, model building and refinement details, as well as the relevant functional assay data for MRGPRX4.

Method S1: Synthesis route and characterization for MRGPRX4 antagonist compounds

## Supplementary Fig. S1

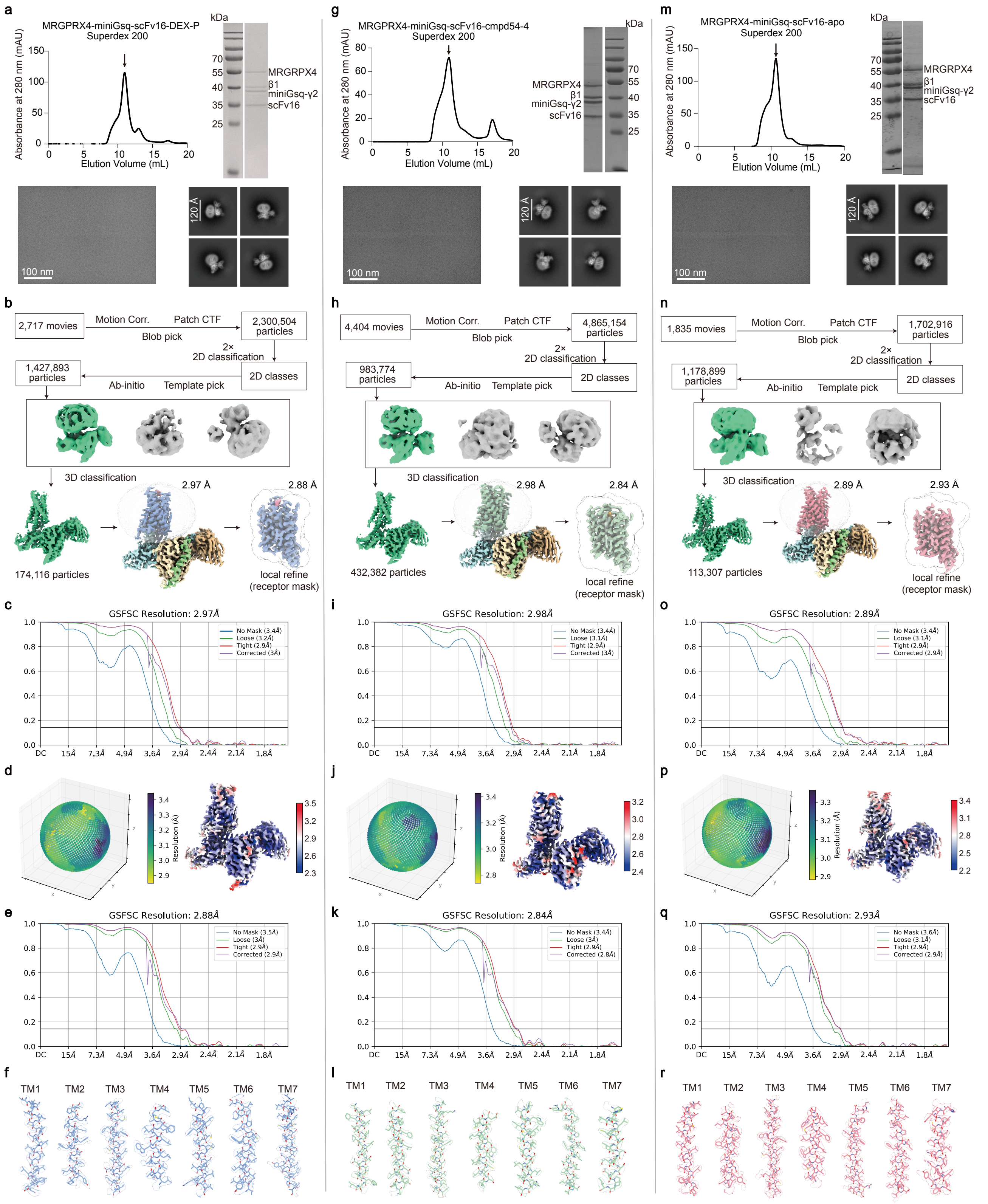


**Fig. S1: Single-particle reconstruction of DEX-P-MRGPRX4-miniGsqβ1γ2-scFv16, cmpd54-4-MRGPRX4-miniGsqβ1γ2-scFv16 and apo-MRGPRX4-miniGsqβ1γ2-scFv16 complex. a, g, m**

Representative size exclusion chromatogram (SEC) profile, SDS-PAGE analysis, cryo-EM micrograph (scale bar: 100 nm) and two-dimensional class averages (scale bar: 120 Å) of DEX-P-MRGPRX4-miniGsqβ1γ2-scFv16 (**a**), cmpd54-4-MRGPRX4-miniGsqβ1γ2-scFv16 (**g**) and apo-MRGPRX4-miniGsqβ1γ2-scFv16 (**m**). **b, h, n** Cryo-EM data processing flow chart of DEX-P-MRGPRX4-miniGsqβ1γ2-scFv16 (**b**), cmpd54-4-MRGPRX4-miniGsqβ1γ2-scFv16 (**h**) and apo-MRGPRX4-miniGsqβ1γ2-scFv16 (**n**). **c, i, o** GSFSC plot calculated by non-uniform refinement from cryoSPARC of DEX-P-MRGPRX4-miniGsqβ1γ2-scFv16. (**c**), cmpd54-4-MRGPRX4-miniGsqβ1γ2-scFv16 (**i**) and apo-MRGPRX4-miniGsqβ1γ2-scFv16 (**o**). **d, j, p** Direction distribution using cFSC vs. conical axis with a gold-standard cut-off of 0.143 (left) and local resolution estimation heatmap of (right) of DEX-P-MRGPRX4-miniGsqβ1γ2-scFv16 (**d**), cmpd54-4-MRGPRX4-miniGsqβ1γ2-scFv16 (**j**) and apo-MRGPRX4-miniGsqβ1γ2-scFv16 (**p**). **e, k, q** GSFSC plot calculated by local refinement from cryoSPARC of DEX-P-MRGPRX4 (**e**), cmpd54-4-MRGPRX4 (**k**) and apo-MRGPRX4 (**q**). **f, l, r** Local cryo-EM maps (silhouetted) of TM1-7 for DEX-P-MRGPRX4 (**f**), cmpd54-4-MRGPRX4 (**l**) and apo-MRGPRX4 (**r**).

## Supplementary Fig. S2

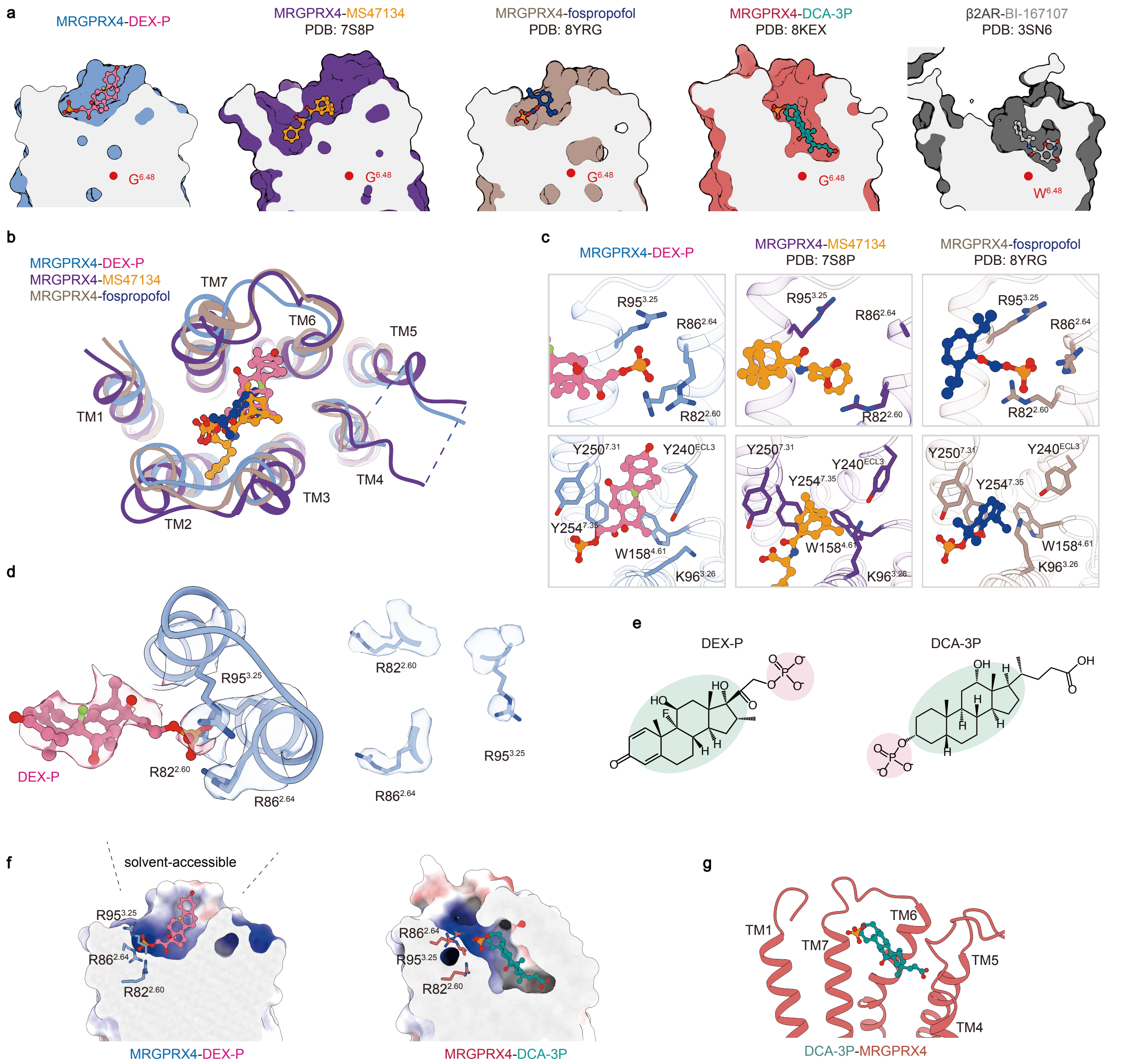


**Fig. S2: Structural features of MRGPRX4 bound with agonists. a** Cut-away views of the ligand-binding pockets in MRGPRX4 structures bound to DEX-P, MS47134 (PDB: 7S8P), fospropofol (PDB: 8YRG), and DCA-3P (PDB: 8KEX), and in the BI-167107-bound $\beta_2$AR structure (PDB: 3SN6). **b** Structural comparison of the DEX-P-MRGPRX4, MS47134-MRGPRX4 and fospropofol-MRGPRX4. Receptors are shown as cartoon. Ligands are shown as ball and stick. **c** The key amino acids involved in agonist recognition are conserved across the DEX-P-bound, MS47134-bound and fospropofol-

bound structures. **d** Electron density map for the DEX-P ligand and its phosphate-binding pocket, contoured level at 0.1. **e** DEX-P and DCA-3P share a common steroid core (green) and an acidic group (pink) that engages the basic pocket of the receptor. **f** Cut-away views of the ligand-binding pockets in the DEX-P-bound (left) and DCA-3P-bound (right) receptor structures, colored by electrostatic potential (blue: positive, red: negative). Key basic residues within the pocket are shown as sticks. **g** Binding pocket of DCA-3P.

## Supplementary Fig. S3

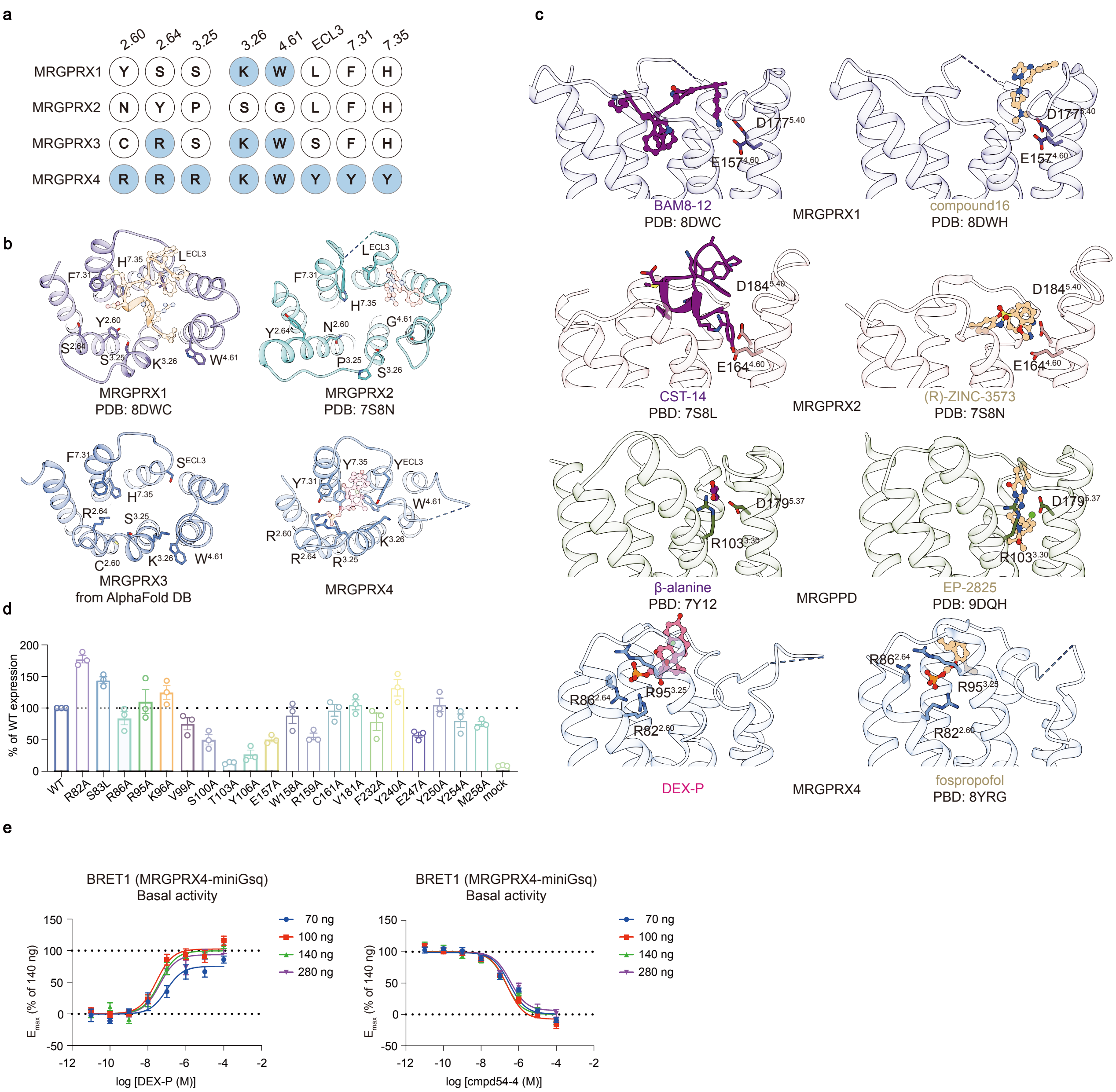


**Fig. S3: Unique structural features of MRGPRX4 in MRGPRs family.** **a** Sequence alignment of the key residues in DEX-P binding pocket. **b** Structural comparison of the DEX-P binding site across MRGPRX family receptors. Amino acid residues at positions equivalent to the DEX-P binding site in MRGPRX4 are shown as sticks for each family member. **c** Comparative structural analysis of agonists bound to MRGPR family members. The following structures were analyzed: BAM8-12–MRGPRX1 (PDB: 8DWC), compound 16–MRGPRX1 (PDB: 8DWH), CST-14–MRGPRX2 (PDB: 7S8L), (R)-

ZINC-3573–MRGPRX2 (PDB: 7S8N), β-alanine–MRGPRD (PDB: 7Y12), EP-2825–MRGPRD (PDB: 9DQH), and fospropofol–MRGPRX4 (PDB: 8YRG). Key residues forming hydrogen bonds or ionic interactions with the ligands are highlighted as sticks. Endogenous and synthetic agonists are colored purple and wheat, respectively. The receptor is depicted as a transparent cartoon. **d** Quantification of MRGPRX4 mutant expression levels by enzyme-linked immunosorbent assay (ELISA). Data are shown as the percentage of WT expression. Data are means ± SEM of three independent experiments performed in duplicate. These data are related to Supplementary Tables S6, S7. **e** Dose–response curves for DEX-P and cmpd54-4 across a range of receptor expression levels, assessed by miniGsq modulation in BRET1 assays. Data are means ± SEM of three independent experiments performed in duplicate. These data are related to Supplementary Tables S6, S7.

## Supplementary Fig. S4

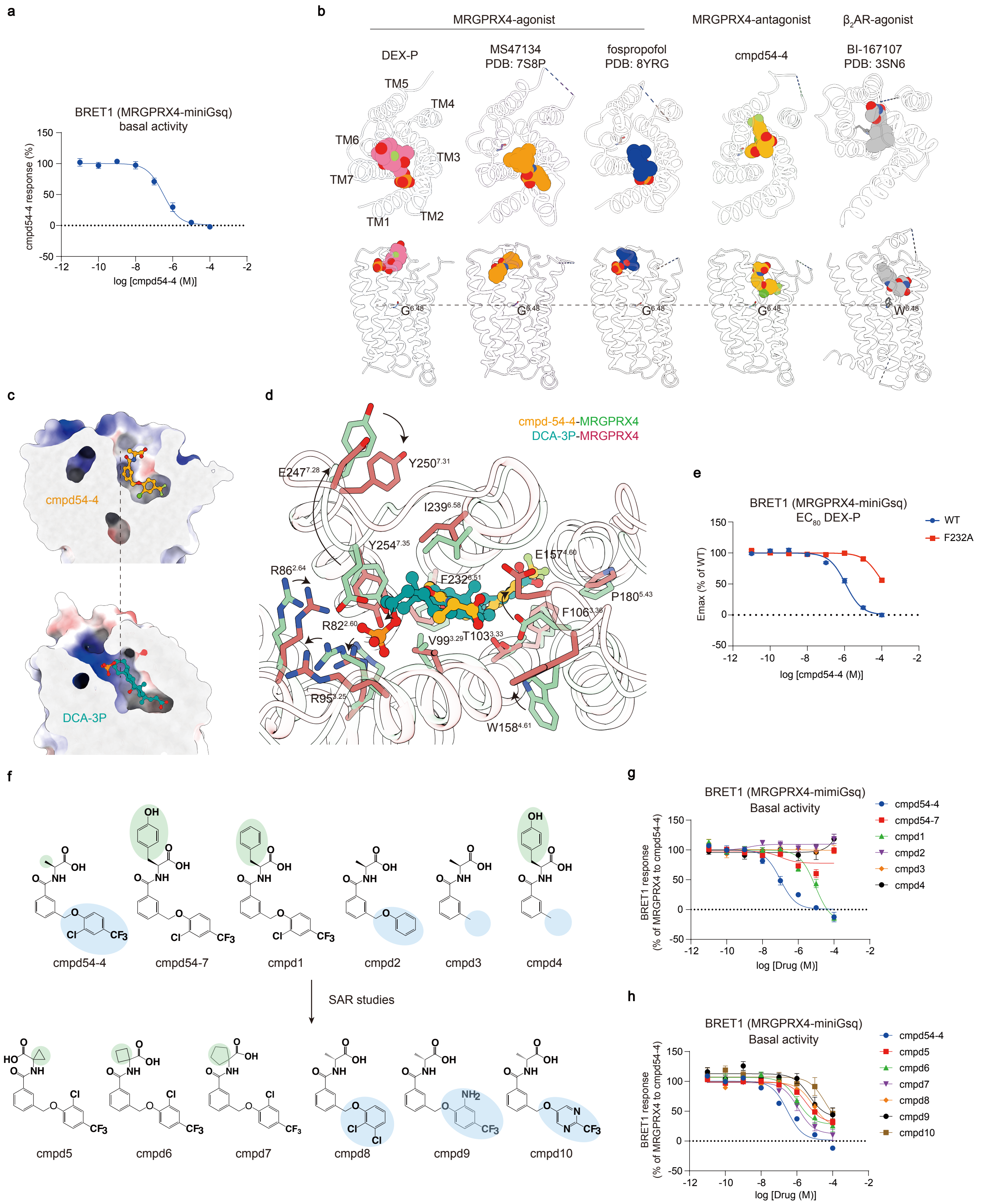


**Fig. S4: Structural features of MRGPRX4 bound with antagonist cmpd54-4. a** cmpd54-4 exhibited inverse agonist activity for MRGPRX4. A concentration-response curve was obtained in the

BRET1 assay by adding only serially diluted inverse agonist. Data are means ± SEM of three independent experiments performed in duplicate. These data are related to Supplementary Table S2. **b** Structural alignment shows that the agonists DEX-P, MS47134 (PDB: 7S8P), and fospropofol (PDB: 8YRG) share a common binding site in MRGPRX4. In contrast, the antagonist cmpd54-4 binds at a distinct location, which instead closely matches the position of the full agonist BI-167107 in $\beta_2$AR (PDB: 3SN6). Receptors are shown as cartoon diagrams; ligands are depicted as spheres. **c** Electrostatic surface potential for the MRGPRX4 receptor in complex with cmpd54-4 and DCA-3P. Surfaces are colored red for negative and blue for positive potential. **d** Structural comparison of the binding site of cmpd54-4 and DCA-3P in MRGPRX4. **e** The effect of the F232$^{6.51}$A mutation on cmpd54-4 activity was assessed in the presence of DEX-P at its EC80 concentration. Response data were normalized to the wild-type (WT) receptor. Data are means ± SEM of three independent experiments performed in duplicate. These data are related to Supplementary Tables S3, S6, S7. **f** Modification of functional groups in methyl from alanyl moiety (green) and 2-chloro-4-(trifluoromethyl)phenoxy group (blue) of cmpd54-4. **g, h** The modified molecules exhibited decreased antagonist potency at MRGPRX4 in the BRET1 assay compared with cmpd54-4. Response data were normalized to the response induced by cmpd54-4. Data are means ± SEM of three independent experiments performed in duplicate. These data are related to Supplementary Table S4.

## Supplementary Fig. S5

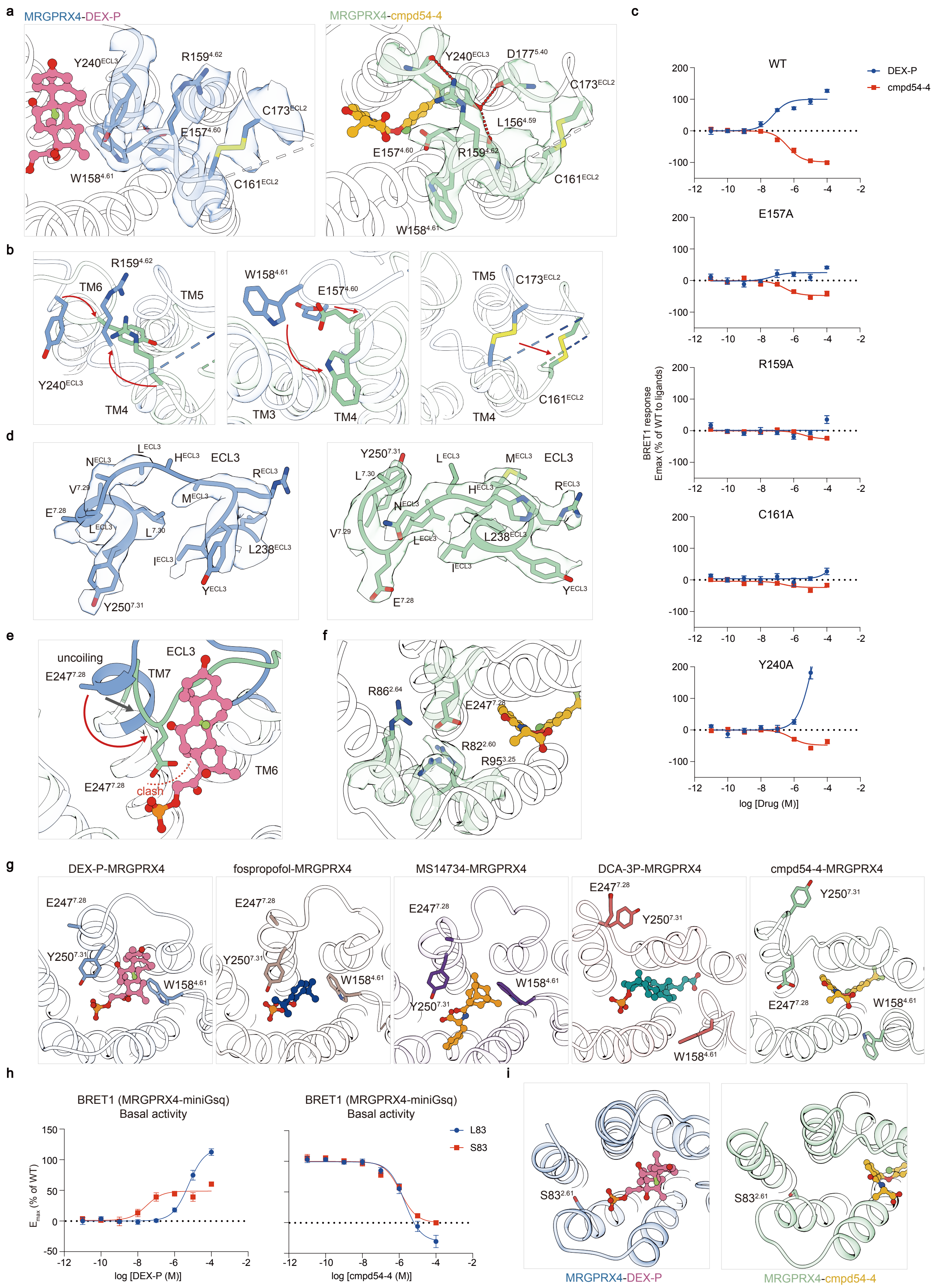

**Fig. S5: Structural comparison of MRGPRX4 bound to DEX-P versus cmpd54-4. a** Distribution of key residues around the ECL2 of the receptor in DEX-P-bound structure and cmpd54-4-bound structure. **b** Conformational heterogeneity of key residues around the ECL2 between DEX-P (blue) and cmpd54-4 (green). Red arrows indicate the transition from the DEX-P-bound to the cmpd54-4-bound state. **c** Functional response curves for DEX-P and cmp54-4 following alanine substitution of key residues. Data are means ± SEM of three independent experiments performed in duplicate. These data are related to Supplementary Tables S3, S6, S7. **d** Structural alignment reveals that the extracellular segment of TM7 undergoes uncoiling upon transitioning from the DEX-P-bound to the cmpd54-4-bound state. In this rearrangement, residue $E247^{7.28}$ flips into the orthosteric pocket, a movement that would result in a steric clash with DEX-P, thereby physically occluding agonist binding. The conformational transitions of the TM7 segment (dark gray arrows) and of $E247^{7.28}$ (red arrow) are indicated. **e** $E247^{7.28}$ occupies the orthosteric site in the cmpd54-4-bound structure, forming polar interactions with three arginine residues. **f** Functional characterization of the $L83^{2.61}$ and $S83^{2.61}$ variants in response to the agonist DEX-P and the antagonist cmpd54-4. Response data were normalized to the S83 receptor. Data are means ± SEM of three independent experiments performed in duplicate. These data are related to Supplementary Tables S5, S6, S7. **g** Top view of residue 83 in complex with DEX-P and cmpd54-4.

## Supplementary Fig. S6

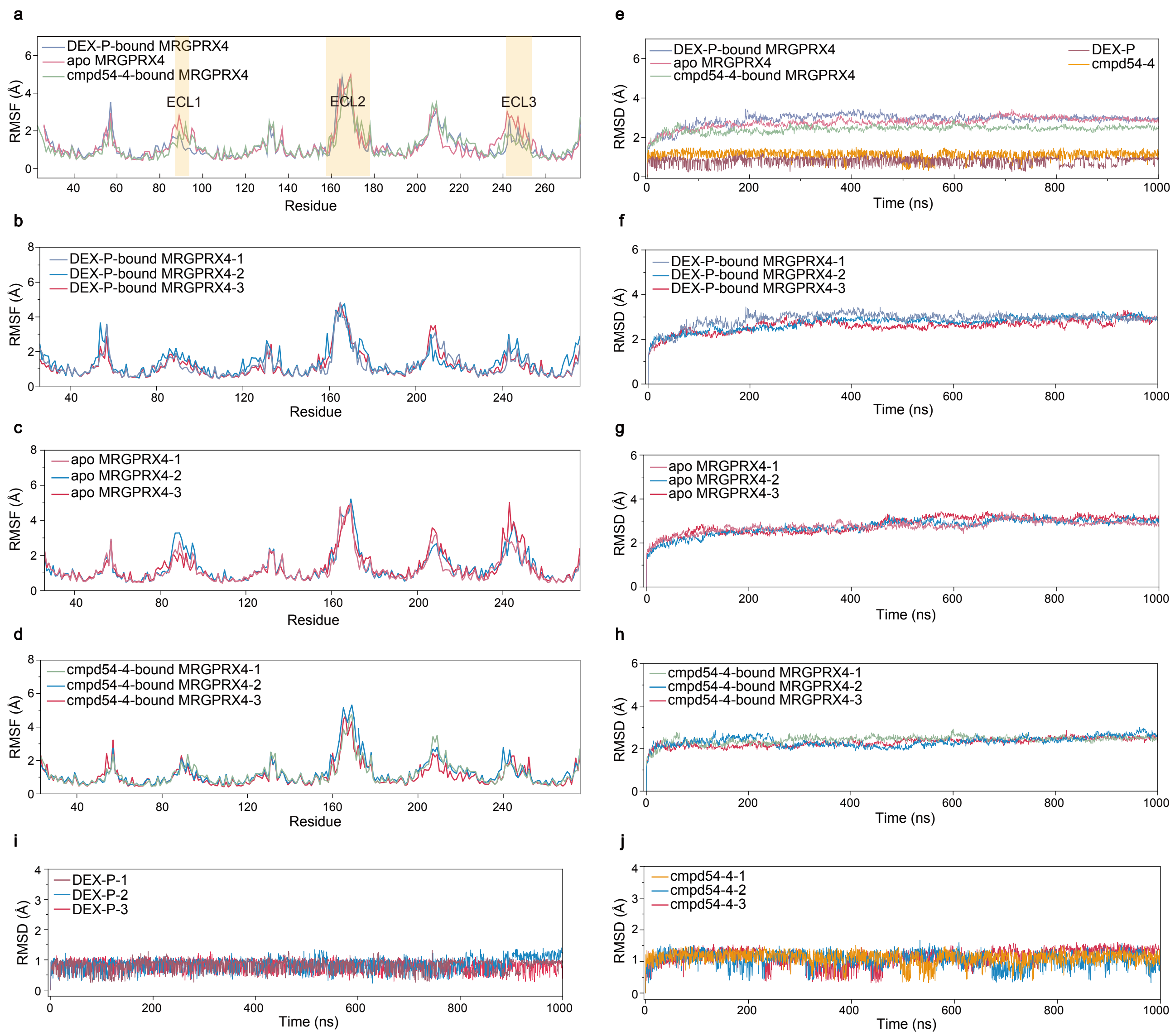


**Fig. S6: Molecular dynamics simulations of MRGPRX4 in three conformational states. a–d** RMSF analyses of the receptor backbone. Overlay of the three states **(a).** Individual trajectories for the DEX-P-bound **(b)**, apo **(c)**, and cmpd54-4-bound **(d)** states are also shown (each from three independent repeats). **e–j** RMSD analyses. Overlay of all RMSD trajectories: the Cα atoms of the three-state structures and the two ligands (DEX-P and cmpd54-4, **e**). Individual receptor RMSD plots for the DEX-P–bound **(f)**, apo **(g)**, and cmpd54-4–bound **(h)** states. RMSD of the ligand DEX-P **(i)** and cmpd54-4 **(j)**, calculated relative to their initial docking poses. All data are derived from equilibrium

molecular dynamics simulations.

**Supplementary Fig. S7**

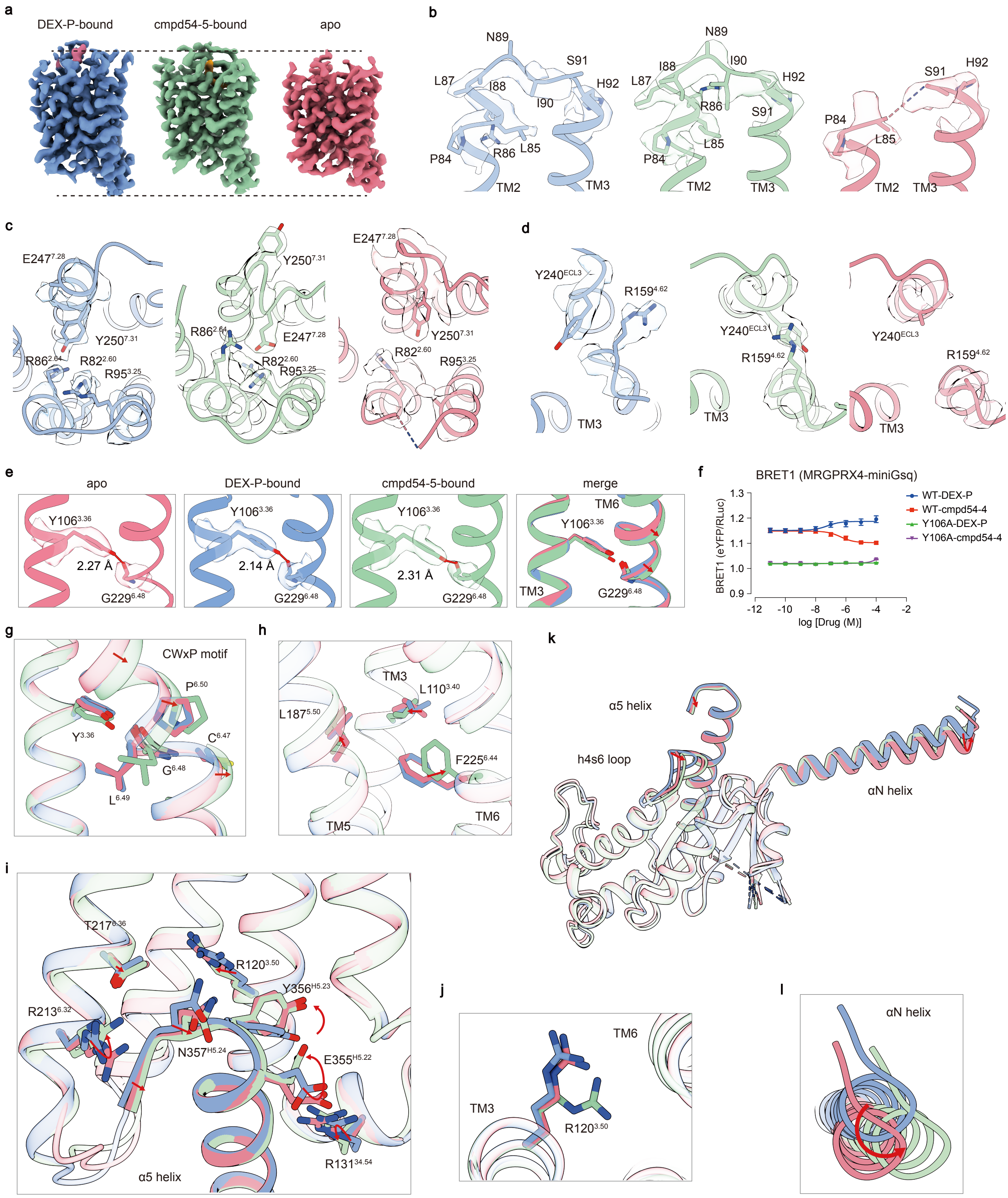


**Fig. S7: Comparative analysis of the three structural states. a** Comparison of the electron density maps among the three states revealed that the apo receptor exhibits weaker density at its extracellular

sides. **b-d** The structural alignment of key residues across three receptor states. Several key amino acids exhibit poorly defined or missing electron density in the apo state structure: within the ECL1 (residues 85-91) (**b**), within the agonist-binding pocket as $R86^{2.64}$, $R95^{3.25}$ and $E247^{7.28}$ (**c**); within the antagonist-binding pocket as $R159^{4.62}$ and $Y240^{ECL3}$ (**d**). The corresponding electron density for these regions is depicted in translucent colors. **e** Variation in hydrogen-bond distance between $Y106^{3.36}$ and $G229^{6.48}$ across the three states correlates with a subtle displacement of TM6. **f** BRET1 dose-response curves for DEX-P and cmpd54-4 at the Y106A mutant compared to WT MRGPRX4. Data are means ± SEM of three independent experiments performed in duplicate. These data are related to Supplementary Tables S3, S6, S7. **g** Conformational changes in the CWxP motif (TM6) among the three states. **h** Conformational changes in the PIF motif among the three states. **i** Differences in the intracellular G-protein-coupling pocket across the three states. Red arrows illustrate the conformational transition of the receptor from the DEX-P-bound state to the apo state, and finally to the cmpd54-4-bound state. **j** Distinct side-chain conformations of the conserved residue $R120^{3.50}$ in the three structures. **k** Superposition of the miniGsq protein in the three complexes, showing its overall positional shift. **l** Close-up view of the miniGsq α5 helix in the three complexes. Red arrows illustrate the conformational transition of the receptor from the DEX-P-bound state to the apo state, and finally to the cmpd54-4-bound state.

**Supplementary Table S1: Cryo-EM data collection, model refinement, and validation statistics.**

| | DEX-P-MPRGX4-miniGsq-scFv16 | DEX-P-MPRGX4 (local) | cmpd54-4-MPRGX4-miniGsq-scFv16 | cmpd54-4-MPRGX4 (local) | Apo-MPRGX4-miniGsq-scFv16 | Apo-MPRGX4 (local) |
|---|---|---|---|---|---|---|
| **Data collection and processing** | | | | | | |
| Magnification | 105,000 | 105,000 | 105,000 | 105,000 | 105,000 | 105,000 |
| Voltage (kV) | 300 | 300 | 300 | 300 | 300 | 300 |
| Electron exposure (e–/Å$^2$) | 50 | 50 | 50 | 50 | 50 | 50 |
| Number of movies used | 2,717 | 2,717 | 4,404 | 4,404 | 1,835 | 1,835 |
| Defocus mean (SD) µm[1] | 1.6 (0.8) | 1.6 (0.8) | 1.6 (0.8) | 1.6 (0.8) | 1.6 (0.8) | 1.6 (0.8) |
| Pixel size (Å) | 0.82 | 0.82 | 0.82 | 0.82 | 0.82 | 0.82 |
| Symmetry imposed | C1 | C1 | C1 | C1 | C1 | C1 |
| Initial particle images (no.) | 2,300,504 | 2,300,504 | 4,865,154 | 4,865,154 | 1,702,916 | 1,702,916 |
| Final particle images (no.) | 174,116 | 174,116 | 432,382 | 432,382 | 113,307 | 113,307 |
| Map resolution (Å)[2] | 2.97 | 2.88 | 2.98 | 2.84 | 2.89 | 2.93 |
| FSC threshold | 0.143 | 0.143 | 0.143 | 0.143 | 0.143 | 0.143 |
| **Refinement** | | | | | | |
| Initial model used (PDB code) | 8YRG | 8YRG | 8YRG | 8YRG | 8YRG | 8YRG |
| Map sharpening *B* factor (Å$^2$) | -101.5 | -104.1 | -114 | -110.3 | -76.4 | -88.8 |
| Model composition | | | | | | |
| Non-hydrogen atoms | 8099 | 1862 | 8141 | 1907 | 8021 | 1787 |
| Protein residues | 1086 | 245 | 1085 | 224 | 1077 | 236 |
| Ligands | 1 | 1 | 1 | 1 | 0 | 0 |
| *B* factors (Å$^2$) | | | | | | |
| Protein | 28.12 | 43.20 | 40.15 | 47.49 | 49.80 | 48.77 |
| Ligand | 62.22 | 75.84 | 28.45 | 49.62 | / | / |
| R.m.s.d deviations | | | | | | |
| Bond lengths (Å) | 0.005 | 0.007 | 0.005 | 0.006 | 0.005 | 0.006 |
| Bond angles (°) | 0.660 | 0.734 | 0.640 | 0.709 | 0.675 | 0.707 |
| Validation | | | | | | |
| MolProbity score | 1.91 | 2.13 | 1.89 | 1.61 | 1.90 | 1.94 |
| Clashscore | 13.46 | 11.67 | 12.83 | 8.34 | 13.61 | 10.85 |
| Poor rotamers (%) | 0.00 | 0.00 | 0.00 | 0.00 | 0.00 | 0.00 |
| Ramachandran plot | | | | | | |
| Favored (%) | 95.97 | 90.04 | 96.06 | 97.08 | 96.22 | 94.35 |
| Allowed (%) | 4.03 | 9.96 | 3.94 | 2.92 | 3.78 | 5.65 |

| | | | | | | |
|---|---|---|---|---|---|---|
| Disallowed (%) | 0.00 | 0.00 | 0.00 | 0.00 | 0.00 | 0.00 |

**Supplementary Table S2: Response of MRGPRX4 to ligands measured by tango assay and BRET1 assay.**

| Compound | Tango assay $pEC_{50}$ / $pIC_{50}$ ± SEM | BRET1 assay $pEC_{50}$ / $pIC_{50}$ ± SEM |
|---|---|---|
| DCA | 4.813±0.115 | / |
| DEX-P | 6.172±0.030 | 7.262±0.068 |
| cmpd54-4 | 6.854±0.047 | 6.315±0.182 |

Data from Tango assays (HTLA cells) and BRET1 assays (HEK293T cells) are presented as means ± SEM of three independent experiments, each performed in technical duplicate. These data are related to Figs. 2a, 3a and Supplementary Fig. S4a.

**Supplementary Table S3: BRET-based profiling of wild-type and mutant MRGPRX4 activation by DEX-P and cmp54-4.**

| MRGPRX4-DEX-P | | | | MRGPRX4-cmpd54-4 (basal activity) | | | |
|---|---|---|---|---|---|---|---|
| Pos. | Mutants | $pEC_{50}$ ± SEM | span± SEM | Pos. | Mutants | $pIC_{50}$ ± SEM | span± SEM |
| | WT | 7.262±0.068 | 100.000±0.000 | | WT | 6.315±0.182 | 100.000±0.000 |
| 2.60 | R82A | ND | ND | 3.26 | K96A | ND | ND |
| 2.64 | R86A | ND | ND | 3.29 | V99A | 5.975±0.217 | 57.693±9.796 |
| 3.25 | R95A | ND | ND | 3.30 | S100A | 5.796±0.042 | 70.483±1.901 |
| 3.26 | K96A | ND | ND | 3.33 | T103A | 6.654±0.276 | 44.917±2.481 |
| 4.60 | E157A | 6.247±0.986 | 38.747±9.191 | 3.36 | Y106A | ND | ND |
| 4.61 | W158A | ND | ND | 4.60 | E157A | 6.549±0.172 | 47.237±5.736 |
| 4.62 | R159A | ND | ND | 4.61 | W158A | 5.202±0.092 | 74.390±6.776 |
| ECL2 | C161A | ND | ND | 4.62 | R159A | 5.448±0.174 | 26.210±1.413 |
| ECL3 | Y240A | 4.944±0.105 | 387.433±42.878 | ECL2 | C161A | 8.581±0.566 | 19.497±6.175 |
| ECL3 | E247A | ND | ND | 5.44 | V181A | 5.824±0.176 | 46.510±3.184 |
| 7.31 | Y250A | ND | ND | 6.51 | F232A | ND | ND |
| 7.35 | Y254A | ND | ND | ECL3 | Y240A | 6.183±0.084 | 47.910±3.567 |
| 6.51 | F232A | 5.380±0.070 | 357.667±22.867 | ECL3 | E247A | ND | ND |
| 3.36 | Y106A | ND | ND | 7.31 | Y250A | 6.216±0.059 | 60.983±2.637 |
| / | / | / | / | 7.35 | Y254A | ND | ND |
| / | / | / | / | 7.39 | M258A | 5.76±0.199 | 25.017±6.756 |
| / | / | / | / | 3.36 | Y106A | ND | ND |
| | | | | MRGPRX4-cmpd54-4 ($EC_{80}$ DEX-P) | | | |
| / | / | / | / | | WT | 5.938±0.096 | 100.000±0.000 |
| / | / | / | / | 6.51 | F232A | 3.436±0.898 | ND |

Data are from BRET1 assays in HEK293T cells, presented as means ± SEM of three independent experiments performed in technical duplicate. Span is expressed as top minus bottom, representing the maximal response range, and is calculated as a percentage of the maximal response of the WT receptor to a reference ligand (DEX-P or cmp54-4). ND (not detectable) refers to data where a robust concentration response curve could not be established within the concentration range tested. These data are related to Figs. 2e, 3g-i, 4j and Supplementary Figs. S4e, S5c, S7f.

**Supplementary Table S4: Response of MRGPRX4 to ligands measured by BRET assay.**

| Compound | $pIC_{50}$ ± SEM | span± SEM | Compound | $pIC_{50}$ ± SEM | span ± SEM |
|---|---|---|---|---|---|
| cmpd54-4 | 6.972±0.162 | 100±0.00 | cmpd54-4 | 6.470±0.124 | 100±0.00 |
| cmpd54-7 | 7.644±0.654 | 21.363±2.841 | cmpd5 | 5.483±0.087 | 69.350±5.396 |
| cmpd1 | 5.070±0.124 | 121.367±7.790 | cmpd6 | 5.973±0.027 | 78.863±9.300 |
| cmpd2 | ND | ND | cmpd7 | 5.887±0.166 | 86.633±10.384 |
| cmpd3 | ND | ND | cmpd8 | 5.102±0.125 | 76.177±11.563 |
| cmpd4 | ND | ND | cmpd9 | 5.350±0.111 | 73.453±12.130 |
| / | / | / | cmpd10 | ND | ND |

Data from BRET assay in HEK293T cells are presented as means ± SEM of three independent experiments, each performed in technical duplicate. Span is expressed as top minus bottom, representing the maximal response range, and is calculated as a percentage of the maximal response to the cmp54-4. “ND” indicates that no reliable concentration-response curve was obtained within the tested range. Of note, the positive control cmpd54-4 was tested in two independent assay sets; the data shown here and in the associated table are from these separate experiments and thus differ from values reported earlier (related to Supplementary Fig. S4g-h).

**Supplementary Table S5: BRET-based profiling of S83 and L83 MRGPRX4 activation by DEX-P and cmp54-4.**

| MRGPRX4-DEX-P | | | | MRGPRX4-cmpd54-4 (basal activity) | | | |
|---|---|---|---|---|---|---|---|
| Pos. | Mutants | $pEC_{50}$ ± SEM | span ± SEM | Pos. | Mutants | $pIC_{50}$ ± SEM | span ± SEM |
| 2.61 | S83 | 7.521±0.398 | 100.000±0.000 | 2.61 | S83 | 5.937±0.083 | 100.000±0.000 |
| 2.61 | L83 | 5.228±0.083 | 238.367±5.296 | 2.61 | L83 | 5.720±0.150 | 130.433±14.722 |

Data are from BRET1 assays in HEK293T cells, presented as means ± SEM of three independent experiments performed in technical duplicate. Span is expressed as top minus bottom, representing the maximal response range, and is calculated as a percentage of the maximal response of the S83 receptor to a reference ligand (DEX-P or cmp54-4). These data are related to Supplementary Fig. S5f.

**Supplementary Table S6: Measurement of the cell surface expression of (WT) MRGPRX4 and mutants by elisa.**

| Pos. | Mutants | Means ± SEM | P value |
|---|---|---|---|
| | WT | 100.00 | |
| 2.60 | R82A | 177.60±6.00 | 0.0238 |
| 2.61 | S83L | 144.20±5.36 | 0.0571 |
| 2.64 | R86A | 84.07±10.43 | 0.7547 |
| 3.25 | R95A | 110.60±18.91 | 0.9981 |
| 3.26 | K96A | 125.00±10.75 | 0.4890 |
| 3.29 | V99A | 75.42±9.42 | 0.4196 |
| 3.30 | S100A | 49.86±8.22 | 0.1011 |
| 3.33 | T103A | 13.78±1.13 | 0.0007 |
| 3.36 | Y106A | 27.15±6.70 | 0.0334 |
| 4.60 | E157A | 50.41±3.79 | 0.0232 |
| 4.61 | W158A | 88.67±12.79 | 0.9637 |
| 4.62 | R159A | 55.20±5.27 | 0.0538 |
| 4.64 | C161A | 96.50±9.24 | >0.9999 |
| 5.44 | V181A | 105.20±8.00 | 0.9941 |
| 6.51 | F232A | 78.44±13.74 | 0.7387 |
| ECL3 | Y240A | 132.10±12.91 | 0.4485 |
| ECL3 | E247A | 57.61±4.29 | 0.0403 |
| 7.31 | Y250A | 105.10±10.61 | 0.9995 |
| 7.35 | Y254A | 80.12±10.61 | 0.6280 |
| 7.39 | M258A | 75.15±3.97 | 0.0966 |
| | mock | 8.93±0.51 | 0.0002 |

Data are shown as the percentage of WT expression. HEK293T cells were used. Data are presented as means ± SEM from three independent experiments, each performed in technical duplicates. These statistics are analyzed using one-way ANOVA with Dunnett's multiple-comparison test. ns, no significant, *$p < 0.05$, **$p < 0.01$, ***$p < 0.001$. These data are related to Supplementary Fig. S3d.

**Supplementary Table S7: Measurement the influence of differential cell surface expression levels on the functional responses to DEX-P and cmpd54-4.**

| MRGPRX4-DEX-P | | | MRGPRX4-cmpd54-4 (basal activity) | | |
|---|---|---|---|---|---|
| plasmid dosage for receptor | $pEC_{50}$ ± SEM | span ± SEM | plasmid dosage for receptor | $pIC_{50}$ ± SEM | span ± SEM |
| 70 ng | 6.972±0.129 | 75.917±4.007 | 70 ng | 6.606±0.251 | 98.980±5.612 |
| 100 ng | 7.555±0.082 | 102.637±10.573 | 100 ng | 6.637±0.109 | 107.547±7.943 |
| 140 ng | 7.391±0.173 | 100.000±0.000 | 140 ng | 6.692±0.076 | 100.000±0.000 |
| 280 ng | 7.375±0.078 | 93.893±12.212 | 280 ng | 6.430±0.130 | 93.853±9.886 |

Data are from BRET1 assays in HEK293T cells, presented as means ± SEM of three independent experiments performed in technical duplicate. Span is expressed as top minus bottom, representing the maximal response range, and is calculated as a percentage of the response obtained with 140 ng of receptor transfection. These data are related to Supplementary Fig. S5e.

**Method S1:**

All purchased chemical reagents were used without further purification. The flash reverse-phase C18 column chromatography was conducted using a SepaBean machine U200 instrument and 40-60 μm, 120Å, C18 columns, with solvent A ($H_2O$ with or without 0.1% TFA) and solvent B (methanol) as eluents. A linear gradient was used with 10% of methanol in $H_2O$ (with or without 0.1% TFA) to 100% of methanol. Nuclear Magnetic Resonance (NMR) spectra were acquired on a Bruker AV400 MHz spectrometer with 400 MHz for proton ($^1$H NMR) and 101 MHz for carbon ($^{13}$C NMR); chemical shifts are reported in (δ). Low-resolution electrospray ionization mass spectrometry (ESI-MS) results were acquired in positive ion mode using an Agilent LC/MSMS 1290-6475 with an electrospray ionization (ESI) source. High-resolution mass spectra (HRMS) data were acquired in positive ion mode using an Agilent 6545 LC/Q-TOF with an electrospray ionization (ESI) source. HPLC was used to establish the purity of target compounds. High-performance liquid chromatography (HPLC) spectra were acquired using a Shimadzu LC-20AT system with SPD-20A UV-VIS detector for all the final products. Chromatography was performed on a 4.6×150 mm Agilent Eclipse XDB-C18 5 μm column with water containing 0.1% trifluoroacetic acid as solvent A and acetonitrile as solvent B at a flow rate of 1.0 mL/min. The gradient program was as follows: 10% B (0−2 min), 10−100% B (2−16 min), 100% B (16−18 min), 100−80% B (18−19 min) and 80% B (19−20 min). All final compounds had > 96% purity using the HPLC methods described above. MS47134 was synthesized according to the published procedures[1].

**Synthesis of (3-((2-chloro-4-(trifluoromethyl)phenoxy)methyl)benzoyl)-*L*-alanine (cmpd54-4)**

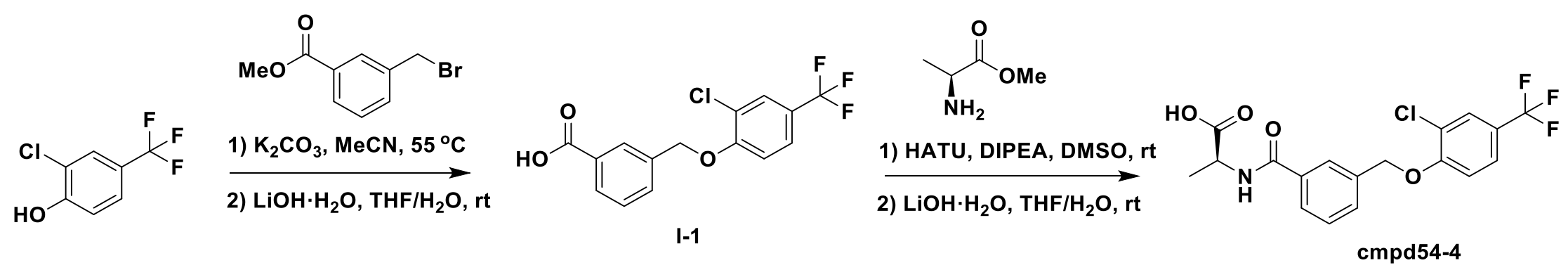


To a solution of the methyl 3-(bromomethyl)benzoate (343.5 mg, 1.5 mmol, 1.0 equiv) in

acetonitrile (MeCN, 4 mL) were added 2-chloro-4-(trifluoromethyl)phenol (324.3 mg, 1.65 mmol, 1.1 equiv) and potassium carbonate ($K_2CO_3$, 310.5 mg, 2.25 mmol, 1.5 equiv) at room temperature. After being stirred at 55 °C for 18 h, the resulting mixture was extracted with ethyl acetate and water. The combined organic phases were washed with water and brine subsequently, and concentrated under vacuum to afford intermediate compound as white solid (423.2 mg, yield 82%). To a solution of the obtained intermediate compound (200 mg, 0.58 mmol, 1.0 equiv) in tetrahydrofuran/water (THF/$H_2O$, 3/1 mL) was added lithium hydroxide monohydrate ($LiOH \cdot H_2O$, 73.1 mg, 1.74 mmol, 3.0 equiv) at room temperature. After being stirred at room temperature for 1 h, the resulting mixture was concentrated under vacuum and purified by reverse-phase C18 flash chromatography (10−100% MeOH in $H_2O$) to afford **3-((2-chloro-4-(trifluoromethyl)phenoxy)methyl)benzoic acid** (**I-1**) as white solid (188.5 mg, yield 98%). $^1$H NMR (400 MHz, Methanol-$d_4$) δ 8.14 (s, 1H), 7.99 (m, *J* = 7.8, 1.5 Hz, 1H), 7.72 – 7.65 (m, 2H), 7.62 – 7.54 (m, 1H), 7.49 (t, *J* = 7.7 Hz, 1H), 7.30 (d, *J* = 8.7 Hz, 1H), 5.31 (s, 2H). ESI-MS: $[M + H]^+$ m/z= 331.2.

To a solution of the **I-1** (33.1 mg, 0.1 mmol, 1.0 equiv) in dimethyl sulfoxide (DMSO, 1.5 mL) were added *L*-Alanine methyl ester hydrochloride (20.9 mg, 0.15 mmol, 1.5 equiv), 1-[bis(dimethylamino)-methylene]-1*H*-1,2,3-triazolo[4,5-*b*]pyridinium 3-oxide hexafluoro-phosphate (HATU, 41.8 mg, 0.11 mmol, 1.1 equiv) and *N,N*-diisopropylethylamine (DIPEA, 38.7 mg, 0.3 mmol, 3.0 equiv) at room temperature. The combined organic phases were washed with water and brine subsequently, and concentrated under vacuum to afford intermediate compound as white oil. To a solution of the above obtained intermediate compound in THF/$H_2O$ (1.2 /0.4 mL) was added $LiOH \cdot H_2O$ (12.6 mg, 0.30 mmol, 3.0 equiv) at room temperature. After being stirred at room temperature for 1 h, the resulting mixture was purified by reverse-phase C18 flash chromatography (10−100% MeOH in $H_2O$) to afford **(3-((2-chloro-4-(trifluoromethyl)phenoxy)methyl)benzoyl)-*L*-alanine** (**cmpd54-4**) as white solid (22.7 mg, yield 57% for two steps). $^1$H NMR (400 MHz, Methanol-$d_4$) δ 7.99 (s, 1H), 7.84 (d, *J* = 7.7 Hz, 1H), 7.72 – 7.65 (m, 2H), 7.60 – 7.47 (m, 2H), 7.31 (d, *J* = 8.6 Hz, 1H), 5.32 (s, 2H), 4.63 (s, 1H), 1.52 (s, 3H). $^{13}$C NMR (101 MHz, Methanol-$d_4$) δ 176.10, 169.76, 158.21, 138.05, 135.81, 131.72, 129.90, 128.30 (q, *J* = 4.0 Hz), 128.26, 127.68, 126.45 (q, *J* = 4.1 Hz), 124.64, 124.39 (q, *J* = 120.7 Hz), 115.10, 71.63, 49.97, 17.50. HRMS (ESI-

TOF) calcd for $C_{18}H_{16}ClF_3NO_4^+$ [M + H]$^+$ 402.0714, found 402.0720.

**Synthesis of (3-((2-chloro-4-(trifluoromethyl)phenoxy)methyl)benzoyl)-*L*-tyrosine (cmpd54-7)**

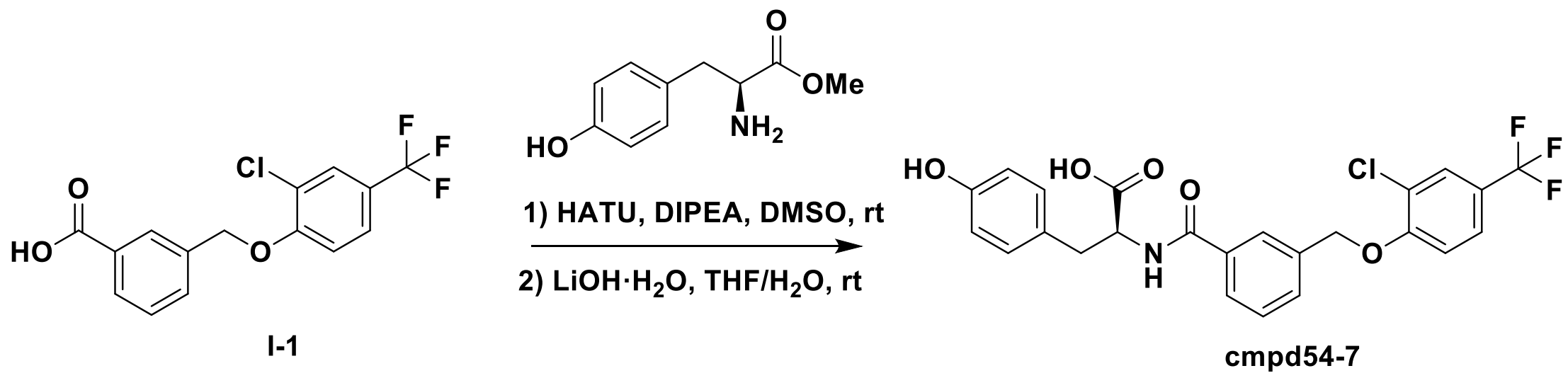


**(3-((2-Chloro-4-(trifluoromethyl)phenoxy)methyl)benzoyl)-*L*-tyrosine (cmpd54-7)** was synthesized following the standard procedure for preparing **cmpd54-4** from methyl *L*-tyrosinate hydrochloride (34.8 mg, 0.15 mmol, 1.5 equiv) and I-1 (33.1 mg, 0.10 mmol, 1.0 equiv), and obtained as white solid (28.3 mg, yield 57% for two steps). $^1$H NMR (400 MHz, Methanol-$d_4$) δ 7.84 (s, 1H), 7.68 (d, $J$ = 2.3 Hz, 2H), 7.62 (d, $J$ = 7.6 Hz, 1H), 7.54 (dd, $J$ = 8.8, 2.3 Hz, 1H), 7.44 (t, $J$ = 7.6 Hz, 1H), 7.25 (d, $J$ = 8.7 Hz, 1H), 7.08 (d, $J$ = 7.8 Hz, 2H), 6.67 (d, $J$ = 7.4 Hz, 2H), 5.24 (s, 2H), 4.78 (s, 1H), 3.25 (d, $J$ = 12.7 Hz, 1H), 3.05 (d, $J$ = 7.9 Hz, 1H). HRMS (ESI-TOF) calcd for $C_{24}H_{20}ClF_3NO_5^+$ [M + H]$^+$ 494.0977, found 494.0986.

**Synthesis of (3-((2-chloro-4-(trifluoromethyl)phenoxy)methyl)benzoyl)-*L*-phenylalanine (cmpd1)**

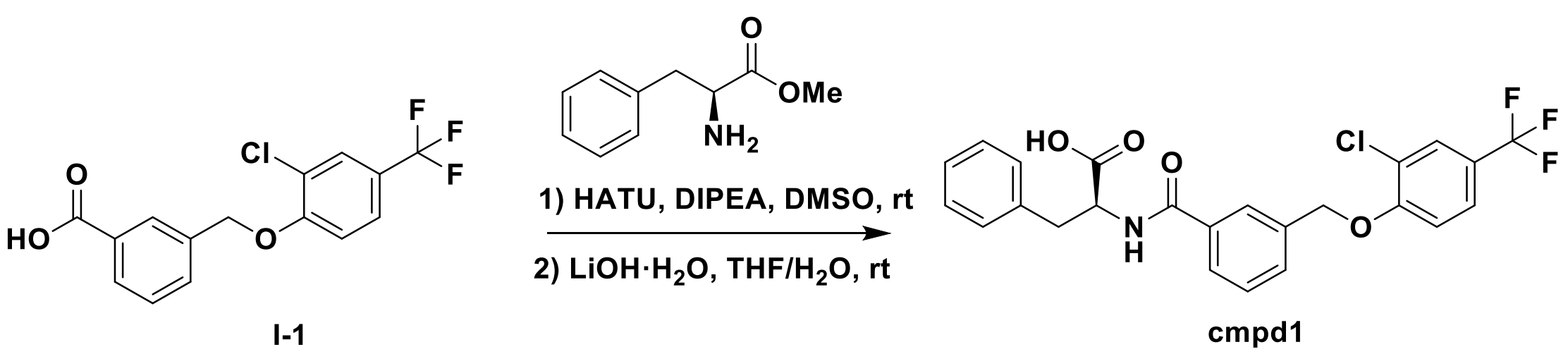


**(3-((2-Chloro-4-(trifluoromethyl)phenoxy)methyl)benzoyl)-*L*-phenylalanine (cmpd1)** was synthesized following the standard procedure for preparing **cmpd54-4** from methyl *L*-phenylalaninate hydrochloride (32.4 mg, 0.15 mmol, 1.5 equiv) and I-1 (33.1 mg, 0.10 mmol, 1.0

equiv), and obtained as white solid (28.4 mg, yield 59% for two steps). $^1$H NMR (400 MHz, Chloroform-*d*) δ 7.84 (s, 1H), 7.69 – 7.60 (m, 3H), 7.51 – 7.42 (m, 2H), 7.34 – 7.27 (m, 3H), 7.24 – 7.19 (m, 2H), 7.01 (d, *J* = 8.6 Hz, 1H), 6.67 (d, *J* = 7.3 Hz, 1H), 5.22 (s, 2H), 5.11 (q, *J* = 6.1 Hz, 1H), 3.40 (dd, *J* = 14.0, 5.5 Hz, 1H), 3.28 (dd, *J* = 14.0, 5.8 Hz, 1H). HRMS (ESI-TOF) calcd for $C_{24}H_{20}ClF_3NO_4^+$ $[M + H]^+$ 478.1027, found 478.1038.

**Synthesis of (3-(phenoxymethyl)benzoyl)-*L*-alanine (cmpd2)**

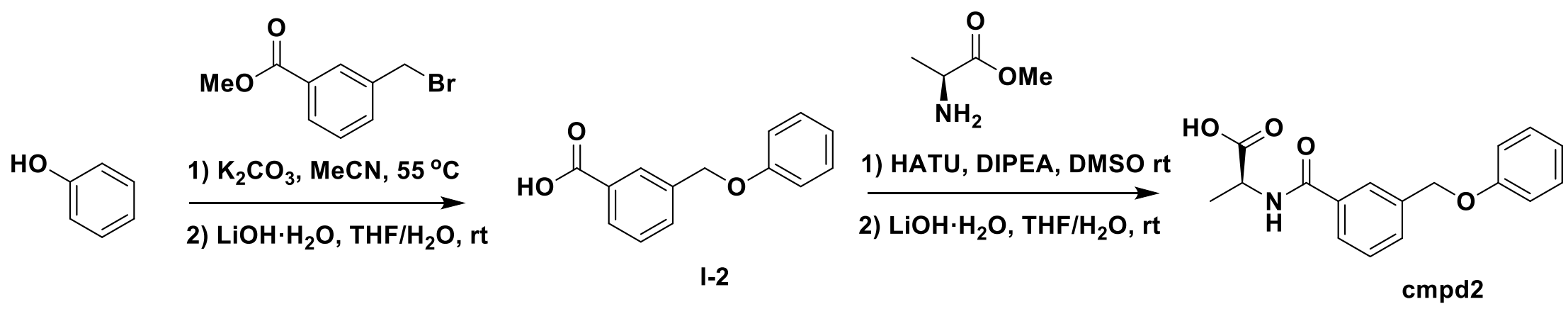


**3-(Phenoxymethyl)benzoic acid (I-2)** was synthesized following the standard procedure for preparing **I-1** from phenol (100.0 mg, 1.06 mmol, 1.0 equiv) and methyl 3-(bromomethyl)benzoate (290.9 mg, 1.27 mmol, 1.2 equiv), and obtained as white solid (178.0 mg, yield 74% for two steps). $^1$H NMR (400 MHz, Methanol-$d_4$) δ 8.11 (s, 1H), 7.98 (d, *J* = 8.0 Hz, 1H), 7.68 (d, *J* = 7.7 Hz, 1H), 7.49 (t, *J* = 7.7 Hz, 1H), 7.28 (t, *J* = 7.8 Hz, 2H), 7.01 (d, *J* = 8.2 Hz, 2H), 6.94 (t, *J* = 7.5 Hz, 1H), 5.14 (s, 2H). ESI-MS: $(M+H)^+$ m/z = 229.1.

**(3-(Phenoxymethyl)benzoyl)-*L*-alanine (cmpd2)** was synthesized following the standard procedure for preparing **cmpd54-4** from *L*-alanine methyl ester hydrochloride (19.5 mg, 0.14 mmol, 1.5 equiv) and I-2 (20.0 mg,0.09 mmol, 1.0 equiv), and obtained as white solid (18.0 mg, yield 67% for two steps).$^1$H NMR (400 MHz, Chloroform-*d*) δ 7.87 (s, 1H), 7.73 (d, *J* = 7.8 Hz, 1H), 7.60 (d, *J* = 7.6 Hz, 1H), 7.44 (t, *J* = 7.8 Hz, 1H), 7.29 (t, *J* = 7.8 Hz, 2H), 6.96 (d, *J* = 7.9 Hz, 3H), 6.78 (d, *J* = 6.9 Hz, 1H), 5.08 (s, 2H), 4.79 (q, *J* = 7.2 Hz, 1H), 1.57 (d, *J* = 7.1 Hz, 3H). HRMS (ESI-TOF) calcd for $C_{17}H_{18}NO_4^+$ $[M + H]^+$ 300.1230, found 300.1230.

**Synthesis of (3-methylbenzoyl)-*L*-alanine (cmpd3)**

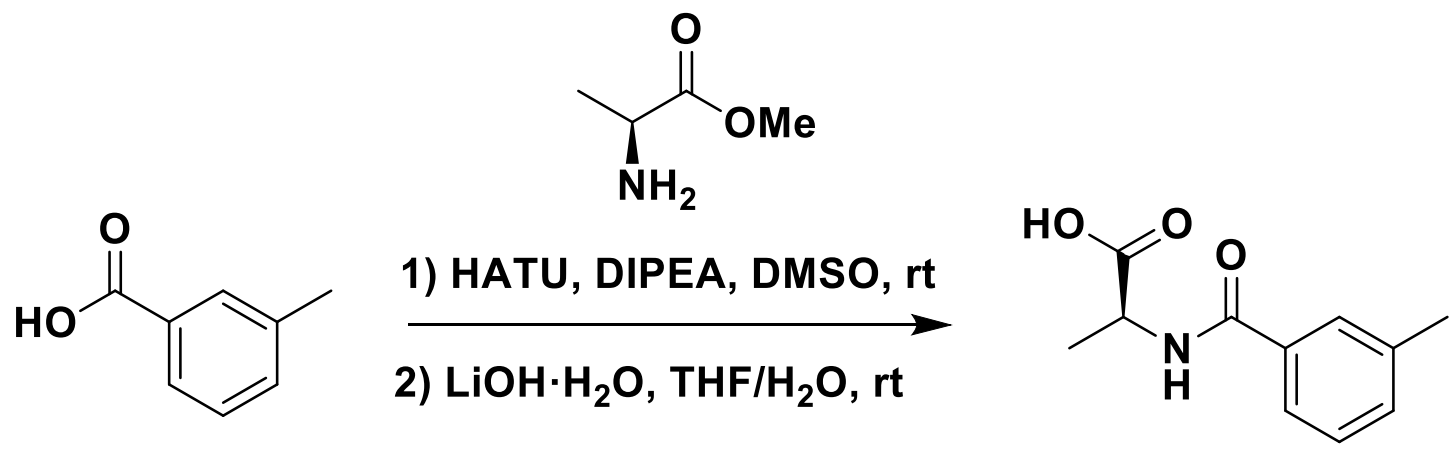


**cmpd3**

**(3-Methylbenzoyl)-*L*-alanine (cmpd3)** was synthesized following the standard procedure for preparing **cmpd54-4** from *L*-alanine methyl ester hydrochloride (32.1 mg,0.23 mmol, 1.5 equiv) and 3-methylbenzoic acid (20.0 mg, 0.15 mmol, 1.0 equiv), and obtained as white solid (25.3 mg, yield 81% for two steps). $^1$H NMR (400 MHz, Methanol-$d_4$) δ 7.68 (s, 1H), 7.66 – 7.60 (m, 1H), 7.33 (d, *J* = 7.1 Hz, 2H), 4.60 (q, *J* = 7.3 Hz, 1H), 2.39 (s, 3H), 1.51 (dd, *J* = 7.3, 1.5 Hz, 3H). HRMS (ESI-TOF) calcd for $C_{11}H_{14}NO_3^+$ [M + H]$^+$ 208.0968, found 208.0969.

**Synthesis of (3-methylbenzoyl)-L-tyrosine (cmpd4)**

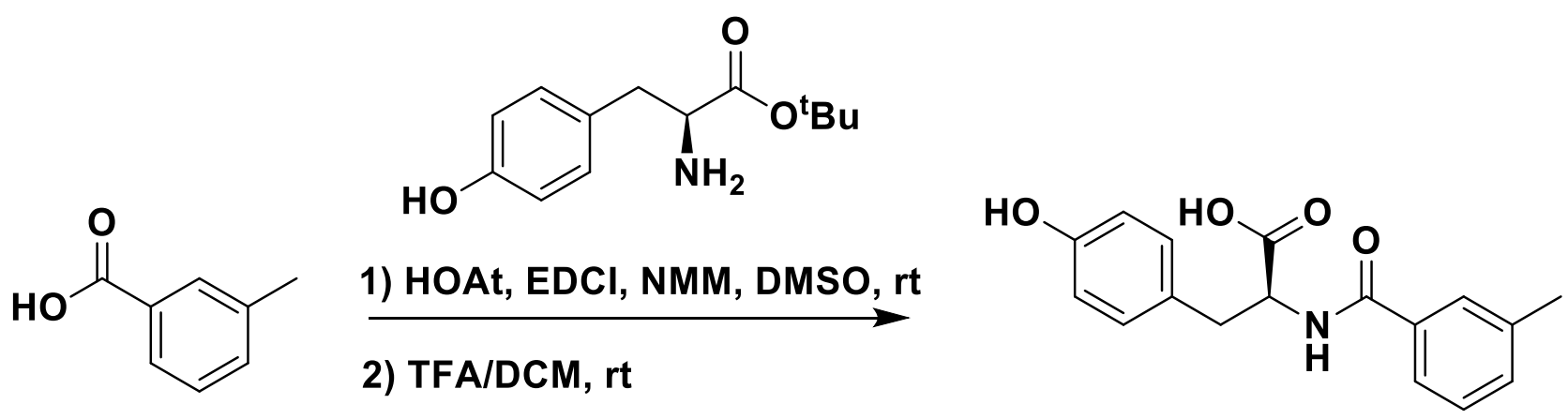


**cmpd4**

To a solution of 3-methylbenzoic acid (20.0 mg, 0.15 mmol, 1.0 equiv) in DMSO (1 mL) were added *tert*-butyl *L*-tyrosinate (54.6 mg, 0.23 mmol, 1.5 equiv), 1-hydroxy-7-azobenzotriazole (HOAt, 40.8 mg, 0.30 mmol, 2.0 equiv), 1-ethyl-3(3-dimethylpropylamine) carbodiimide (EDCI, 57.5 mg, 0.30 mmol, 2.0 equiv) and *N*-methylmorpholine (NMM, 60.7 mg, 0.6 mmol, 4.0 equiv) at room temperature. After being stirred at room temperature for 18 h, the resulting mixture was extracted with ethyl acetate and water. The organic phases were combined and washed with water and brine subsequently. After the organic solvent was evaporated in vacuo, the residue was purified by silica gel column chromatography with petroleum ether / ethyl acetate (PE/EA, 2:1) to afford intermediate compound as white solid. Then to a solution of the above obtained intermediate compound in

dichloromethane (DCM, 1 mL) was added trifluoroacetic acid (TFA, 0.5 mL) at room temperature. After the mixture was stirred at room temperature for 1 h, the resulting mixture was concentrated under vacuum and purified by reverse-phase C18 flash chromatography (10−100% MeOH in $H_2O$) to afford **(3-methylbenzoyl)-*L*-tyrosine (cmpd4)** as white solid (24.6 mg, yield 55% for two steps). $^1$H NMR (400 MHz, Methanol-$d_4$) δ 7.56 – 7.47 (m, 2H), 7.32 (d, *J* = 7.1 Hz, 1H), 7.30 (d, *J* = 7.5 Hz, 1H), 7.09 (d, *J* = 8.1 Hz, 2H), 6.70 (d, *J* = 8.0 Hz, 2H), 4.79 (dd, *J* = 9.1, 5.1 Hz, 1H), 3.23 (dd, *J* = 14.0, 5.1 Hz, 1H), 3.03 (dd, *J* = 14.0, 9.2 Hz, 1H), 2.36 (s, 3H). HRMS (ESI-TOF) calcd for $C_{17}H_{18}NO_4^+$ [M + H]$^+$ 300.1230, found 300.1238.

**Synthesis of 1-(3-((2-chloro-4-(trifluoromethyl)phenoxy)methyl)benzamido)cyclopropane-1-carboxylic acid (cmpd5)**

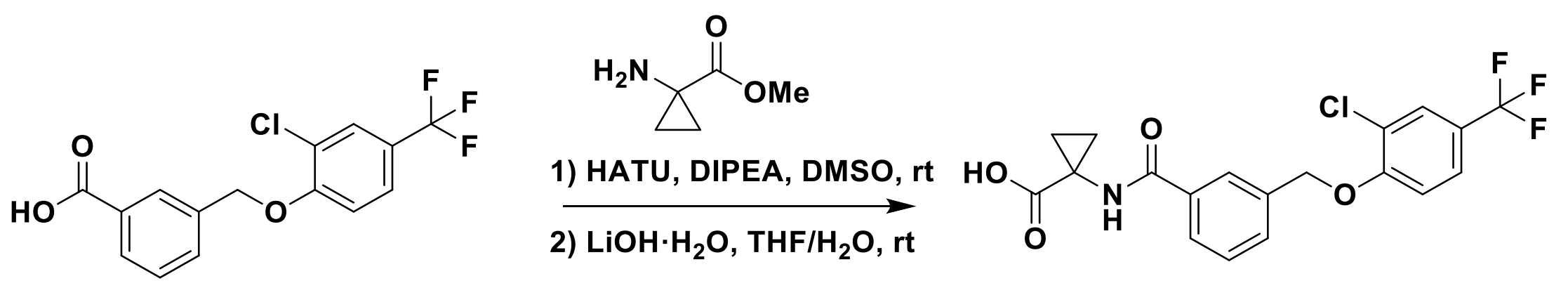


**1-(3-((2-Chloro-4-(trifluoromethyl)phenoxy)methyl)benzamido)cyclopropane-1-carboxylic acid (cmpd5)** was synthesized following the standard procedure for preparing **cmpd54-4** from methyl 1-aminocyclopropanecarboxylate hydrochloride (22.7 mg, 0.15 mmol, 1.5 equiv) and I-1 (33.1 mg, 0.10 mmol, 1.0 equiv), and obtained as white solid (23.1 mg, yield 56% for two steps). $^1$H NMR (400 MHz, Methanol-$d_4$) δ 7.97 (s, 1H), 7.82 (d, J = 7.8 Hz, 1H), 7.69 (d, J = 8.1 Hz, 2H), 7.57 (d, J = 8.7 Hz, 1H), 7.51 (t, J = 7.7 Hz, 1H), 7.31 (d, J = 8.7 Hz, 1H), 5.31 (s, 2H), 1.60 (q, J = 4.8 Hz, 2H), 1.25 (q, J = 4.8 Hz, 2H). HRMS (ESI-TOF) calcd for $C_{19}H_{16}ClF_3NO_4^+$ [M + H]$^+$ 414.0714, found 414.0712.

**Synthesis of 1-(3-((2-chloro-4-(trifluoromethyl)phenoxy)methyl)benzamido)cyclobutane-1-carboxylic acid (cmpd6)**

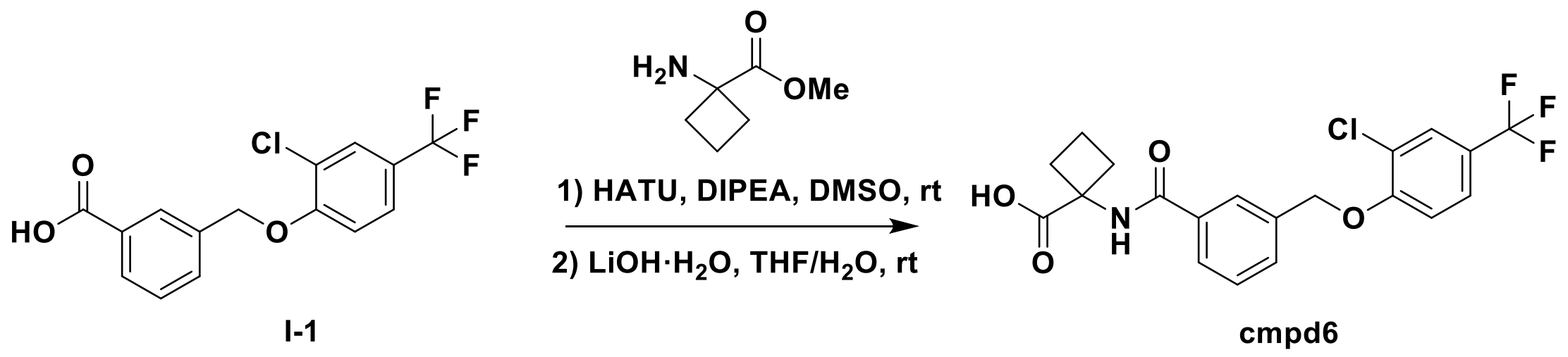

**1-(3-((2-Chloro-4-(trifluoromethyl)phenoxy)methyl)benzamido)cyclobutane-1-carboxylic acid (cmpd6)** was synthesized following the standard procedure for preparing **cmpd54-4** from methyl 1-aminocyclobutane-1-carboxylate (19.4 mg, 0.15 mmol, 1.5 equiv) and I-1 (33.1 mg, 0.10 mmol, 1.0 equiv), and obtained as white solid (24.9 mg, yield 58% for two steps). $^1$H NMR (400 MHz, Methanol-$d_4$) δ 7.98 (s, 1H), 7.84 (d, *J* = 7.8 Hz, 1H), 7.68 (d, *J* = 8.5 Hz, 2H), 7.57 (d, *J* = 8.8 Hz, 1H), 7.51 (t, *J* = 7.7 Hz, 1H), 7.30 (d, *J* = 8.6 Hz, 1H), 5.31 (s, 2H), 2.79 – 2.68 (m, 2H), 2.48 – 2.36 (m, 2H), 2.18 – 1.99 (m, 2H). HRMS (ESI-TOF) calcd for $C_{20}H_{18}ClF_3NO_4^+$ [M + H]$^+$ 428.0871, found 428.0884.

**Synthesis of 1-(3-((2-chloro-4-(trifluoromethyl)phenoxy)methyl)benzamido)cyclopentane-1-carboxylic acid (cmpd7)**

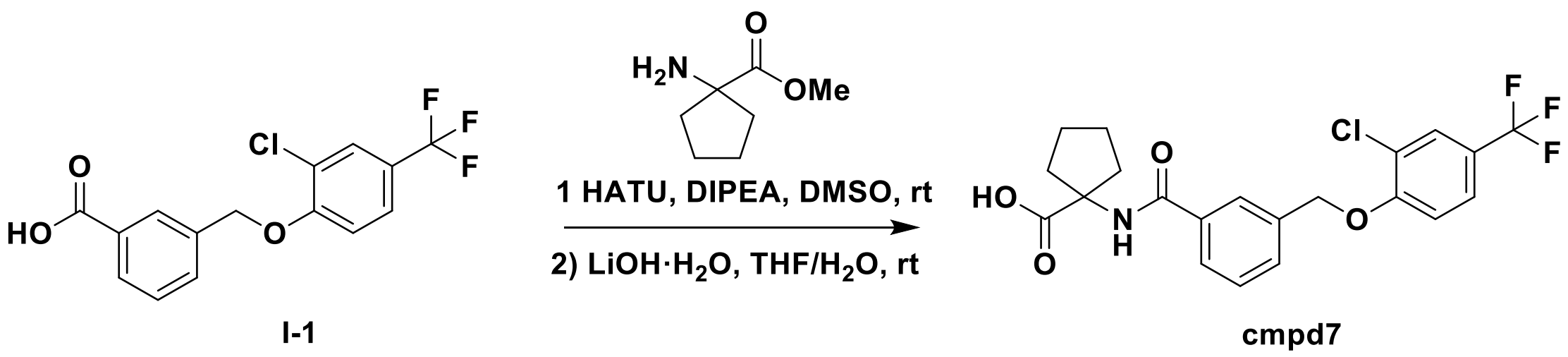

**1-(3-((2-Chloro-4-(trifluoromethyl)phenoxy)methyl)benzamido)cyclopentane-1-carboxylic acid (cmpd7)** was synthesized following the standard procedure for preparing **cmpd54-4** from methyl 1-amino-1-cyclopentanecarboxylate hydrochloride (26.5 mg, 0.15 mmol, 1.5 equiv) and I-1 (33.1 mg, 0.10 mmol, 1.0 equiv),and obtained as white solid (25.8 mg, yield 58% for two steps). $^1$H NMR (400 MHz, Methanol-$d_4$) δ 7.94 (s, 1H), 7.79 (d, *J* = 7.8 Hz, 1H), 7.73 – 7.64 (m, 2H), 7.57 (d, *J* = 8.8 Hz, 1H), 7.50 (t, *J* = 7.8 Hz, 1H), 7.31 (d, *J* = 8.7 Hz, 1H), 5.32 (s, 2H), 2.33 (dt, *J* = 14.2, 7.4 Hz, 2H), 2.13 (dd, *J* = 13.4, 6.3 Hz, 2H), 1.83 (h, *J* = 4.9, 3.9 Hz, 4H). HRMS (ESI-TOF) calcd for $C_{21}H_{20}ClF_3NO_4^+$ [M + H]$^+$ 442.1027, found 442.1037.

**Synthesis of (3-((2,3-dichlorophenoxy)methyl)benzoyl)-*L*-alanine (cmpd8)**

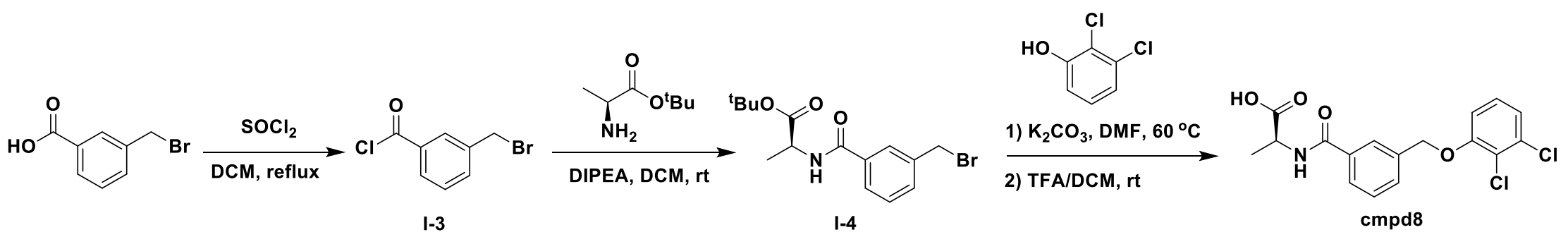

To a solution of 3-(bromomethyl)benzoic acid (300.0 mg, 1.4 mmol, 1.0 equiv) in DCM (5 mL) was added thionyl chloride ($SOCl_2$, 2.5 mL) at 0°C. After being stirred at 60°C for 18h. The resulting mixture was concentrated under vacuum to afford **crude 3-(bromomethyl)benzoyl chloride** (**I-3**) as colorless oil (310.6 mg, yield 95%).

To a solution of (*S*)-2-Aminopropionic acid *tert*-butyl ester hydrochloride (290.7 mg, 1.60 mmol, 1.2 equiv) in DCM (3 mL) were added DIPEA (343.1 mg, 2.66 mmol, 2.0 equiv) and **I-3** (310.6 mg, 1.33 mmol, 1.0 equiv) at 0 °C. After being stirred at room temperature for 20 min, the resulting mixture was concentrated under vacuum and purified by silica gel column chromatography with petroleum ether/ethyl acetate (PE/EA, 6:1) to afford ***tert*-butyl (3-(bromomethyl)benzoyl)-*L*-alaninate** (**I-4**) as white solid (400.0 mg, yield 88%). $^1$H NMR (400 MHz, Chloroform-*d*) δ 7.82 (t, *J* = 1.8 Hz, 1H), 7.74 (dt, *J* = 7.7, 1.5 Hz, 1H), 7.53 (dt, *J* = 7.6, 1.5 Hz, 1H), 7.43 (t, *J* = 7.7 Hz, 1H), 6.83 (d, *J* = 7.3 Hz, 1H), 4.67 (p, *J* = 7.1 Hz, 1H), 4.61 (s, 2H), 1.49 (d, *J* = 7.1 Hz, 12H).

To a solution of **I-4** (30.0 mg, 0.09 mmol, 1.0 equiv) in *N,N*-dimethylformamide (DMF ,1.5 mL) were added 2,3-dichlorophenol (22.8 mg, 0.14 mmol, 1.5 equiv) and $K_2CO_3$ (24.8 mg, 0.18 mmol, 2.0 equiv) at room temperature. After being stirred at 60 °C for 18 h, the resulting mixture was extracted with ethyl acetate and water. The organic phases were combined and washed with water and brine subsequently. The organic phases were concentrated under vacuum and purified by silica gel column chromatography with petroleum ether/ethyl acetate (PE/EA, 6:1) to afford intermediate compound as colorless oil. To a solution of the above obtained intermediate compound in DCM (1 mL) was added TFA (0.5 mL) at room temperature. After being stirred at room temperature for 1 h, the resulting mixture was concentrated under vacuum and purified by reverse-phase C18 flash chromatography (10−100% MeOH in $H_2O$) to afford **(3-((2,3-dichlorophenoxy)methyl)benzoyl)-*L*-alanine** (**cmpd8**) as white solid (20.2 mg, yield 61% for two steps). $^1$H NMR (400 MHz, Methanol-$d_4$)

δ 7.98 (s, 1H), 7.83 (d, $J$ = 7.8 Hz, 1H), 7.69 (d, $J$ = 7.7 Hz, 1H), 7.51 (t, $J$ = 7.8 Hz, 1H), 7.23 (t, $J$ = 8.2 Hz, 1H), 7.12 (t, $J$ = 7.9 Hz, 2H), 5.26 (s, 2H), 4.61 (q, $J$ = 7.3 Hz, 1H), 1.52 (d, $J$ = 7.3 Hz, 3H). HRMS (ESI-TOF) calcd for $C_{17}H_{16}Cl_2NO_4^+$ $[M + H]^+$ 368.0451, found 368.0456.

**Synthesis of (3-((2-amino-4-(trifluoromethyl)phenoxy)methyl)benzoyl)-*L*-alanine (cmpd9)**

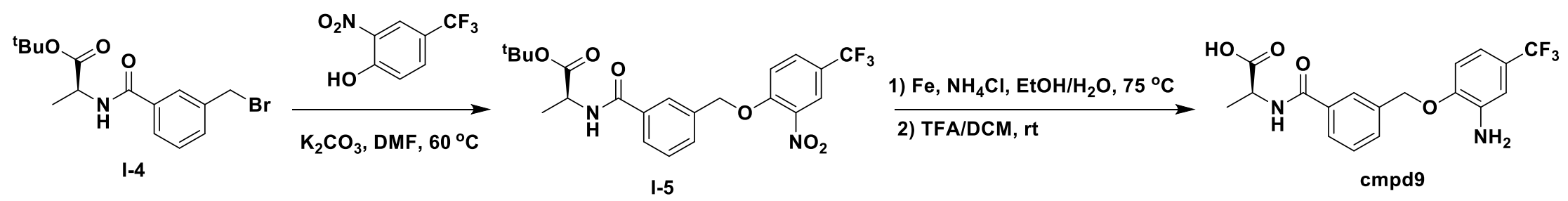


To a solution of **I-4** (30.0 mg, 0.09 mmol, 1.0 equiv) in DMF (1.5 mL) were added 2-nitro-4-(trifluoromethyl)phenol (29.0 mg, 0.14 mmol, 1.5 equiv) and $K_2CO_3$ (24.8 mg, 0.18 mmol, 2.0 equiv) at room temperature. After being stirred at 60 °C for 18 h, the resulting mixture was extracted with ethyl acetate and water. The organic phases were combined and washed with water and brine subsequently. The organic phases were concentrated under vacuum and purified by silica gel column chromatography with petroleum ether/ethyl acetate (PE/EA, 4:1) to afford ***tert*-butyl (3-((2-nitro-4-(trifluoromethyl)phenoxy)methyl)benzoyl)-*L*-alaninate** (**I-5**) as brown oil (31.3 mg, yield 74%). $^1H$ NMR (400 MHz, Chloroform-*d*) δ 8.15 (d, $J$ = 2.3 Hz, 1H), 7.91 (d, $J$ = 2.3 Hz, 1H), 7.77 (dd, $J$ = 8.6, 2.9 Hz, 2H), 7.64 (d, $J$ = 7.7 Hz, 1H), 7.49 (t, $J$ = 7.7 Hz, 1H), 7.23 (d, $J$ = 8.8 Hz, 1H), 6.84 (d, $J$ = 7.2 Hz, 1H), 5.34 (s, 2H), 4.67 (p, $J$ = 7.1 Hz, 1H), 1.50 (d, $J$ = 2.0 Hz, 12H). ESI-MS: $(M+H)^+$ m/z = 469.2

To a solution of **I-5** (31.3 mg, 0.07 mmol, 1.0 equiv) in EtOH/$H_2O$ (1 mL/0.5 mL) were added Fe (19.6 mg, 0.35 mmol, 5.0 equiv), ammonium chloride ($NH_4Cl$, 26.2 mg, 0.49 mmol, 7.0 equiv) at room temperature. After being stirred at 75 °C for 4 h, the reaction mixture was filtered and the filtrate was concentrated under vacuum to afford intermediate compound as brown oil. To a solution of the above obtained intermediate compound in DCM (1 mL) was added TFA (0.5 mL) at room temperature. After being stirred at room temperature for 1 h, the resulting mixture was concentrated under vacuum and purified by reverse-phase C18 flash chromatography (10−100% MeOH in $H_2O$) to afford **(3-((2-amino-4-(trifluoromethyl)phenoxy)methyl)benzoyl)-*L*-alanine (cmpd9)** as brown solid. (19.3 mg, yield 72% for two steps). $^1H$ NMR (400 MHz, Methanol-$d_4$) δ 7.97 (d, $J$ = 1.9 Hz,

1H), 7.81 (d, $J$ = 7.8 Hz, 1H), 7.65 (d, $J$ = 7.7 Hz, 1H), 7.48 (t, $J$ = 7.7 Hz, 1H), 7.29 (d, $J$ = 7.7 Hz, 2H), 7.19 (d, $J$ = 8.4 Hz, 1H), 5.29 (s, 2H), 4.57 (q, $J$ = 7.4 Hz, 1H), 1.48 (d, $J$ = 7.3 Hz, 3H). HRMS (ESI-TOF) calcd for $C_{18}H_{18}F_3N_2O_4^+$ $[M + H]^+$ 383.1213, found 383.1208.

**Synthesis of (3-(((2-(trifluoromethyl)pyrimidin-5-yl)oxy)methyl)benzoyl)-*L*-alanine (cmpd10)**

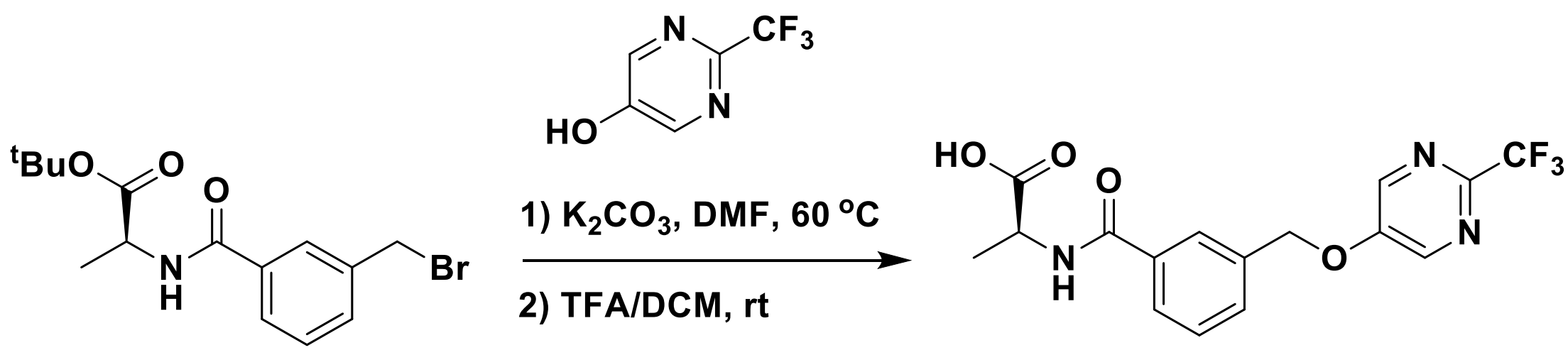


I-4

cmpd10

**(3-(((2-(Trifluoromethyl)pyrimidin-5-yl)oxy)methyl)benzoyl)-*L*-alanine (cmpd10)** was synthesized following the standard procedure for preparing cmpd10 from 2-(trifluoromethyl)pyrimidin-5-ol (23.0 mg, 0.14 mmol, 1.5 equiv) and **I-4** (30.0 mg, 0.09 mmol, 1.0 equiv), and obtained as white solid (23.7 mg, yield 71% for two steps). $^1$H NMR (400 MHz, Methanol-$d_4$) δ 8.70 (s, 2H), 8.00 (t, $J$ = 1.8 Hz, 1H), 7.87 (dt, $J$ = 7.9, 1.5 Hz, 1H), 7.71 – 7.64 (m, 1H), 7.52 (t, $J$ = 7.7 Hz, 1H), 5.39 (s, 2H), 4.61 (q, $J$ = 7.4 Hz, 1H), 1.52 (d, $J$ = 7.3 Hz, 3H). HRMS (ESI-TOF) calcd for $C_{16}H_{15}F_3N_3O_4^+$ $[M + H]^+$ 370.1009, found 370.1013.

## $^1$H-NMR of cmpd54-4 in Methanol-d4

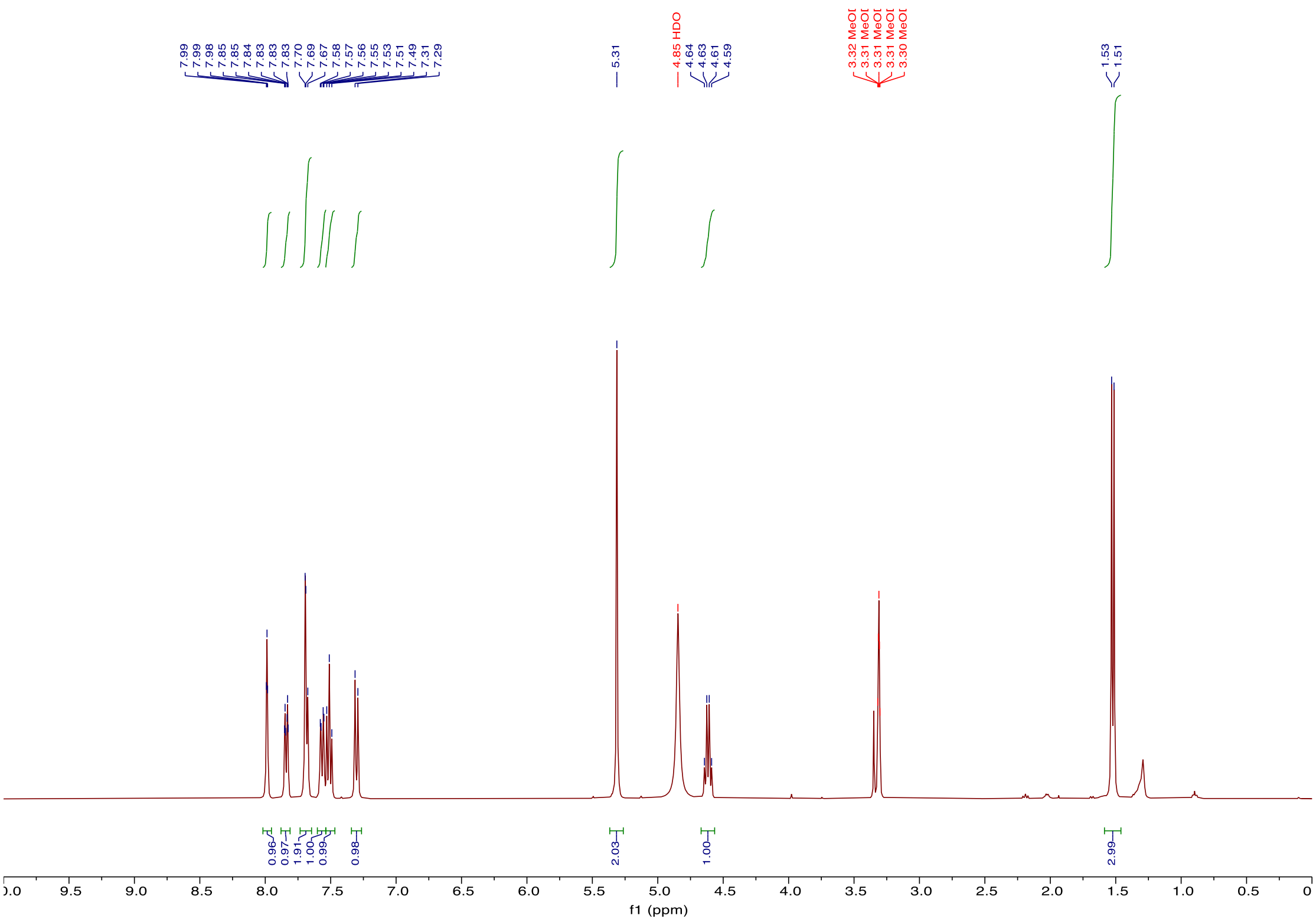

## $^{13}$C-NMR of cmpd54-4 in Methanol-d4

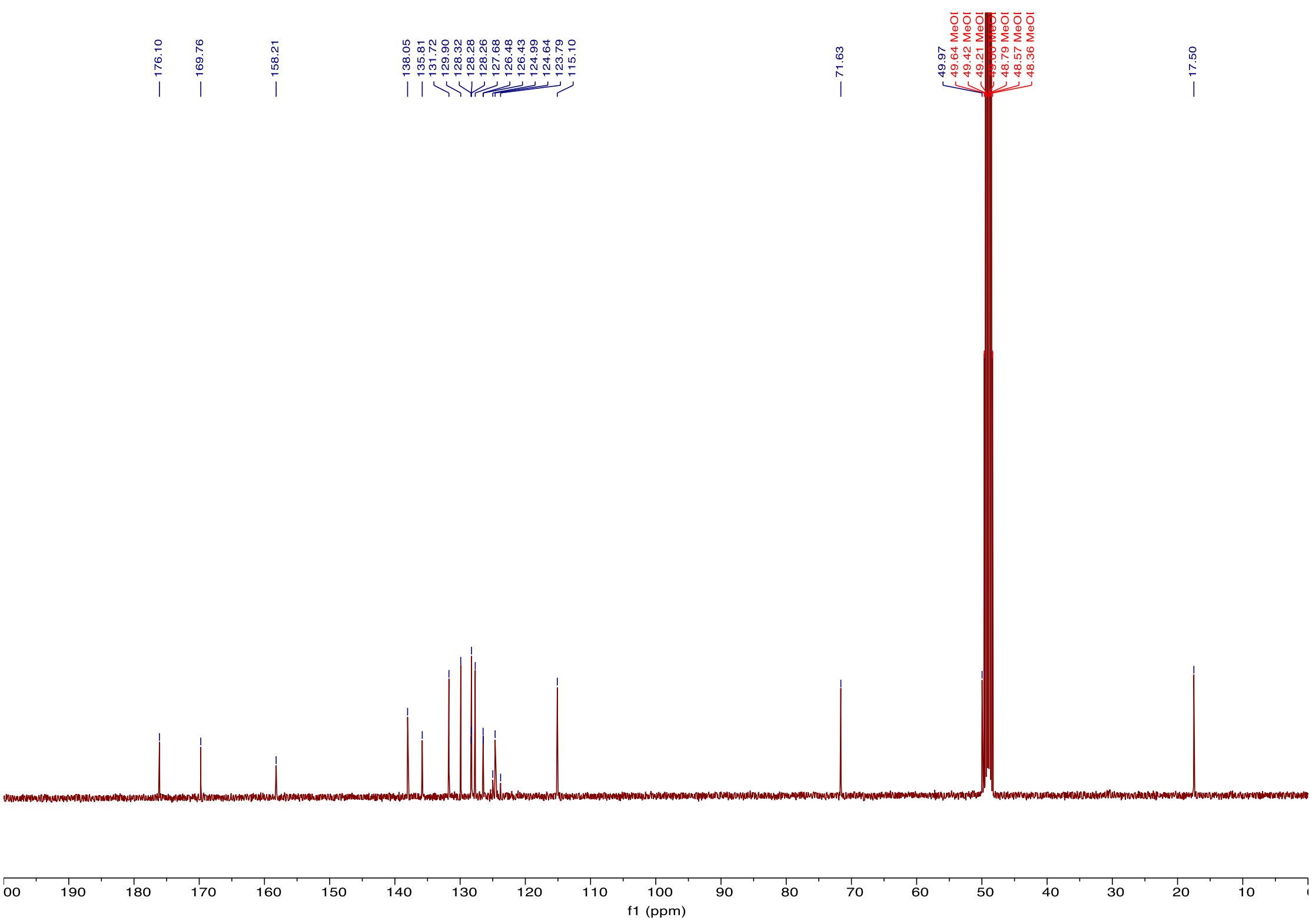

**HRMS spectrum of cmpd54-4**

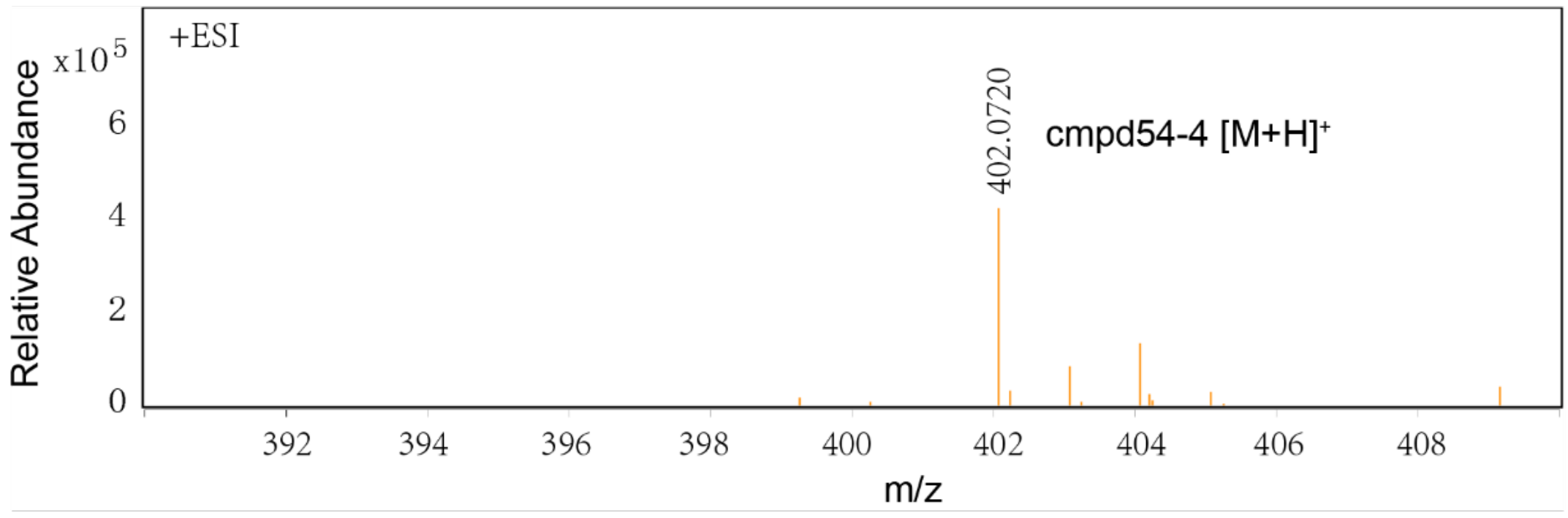


**HPLC spectrum of cmpd54-4**

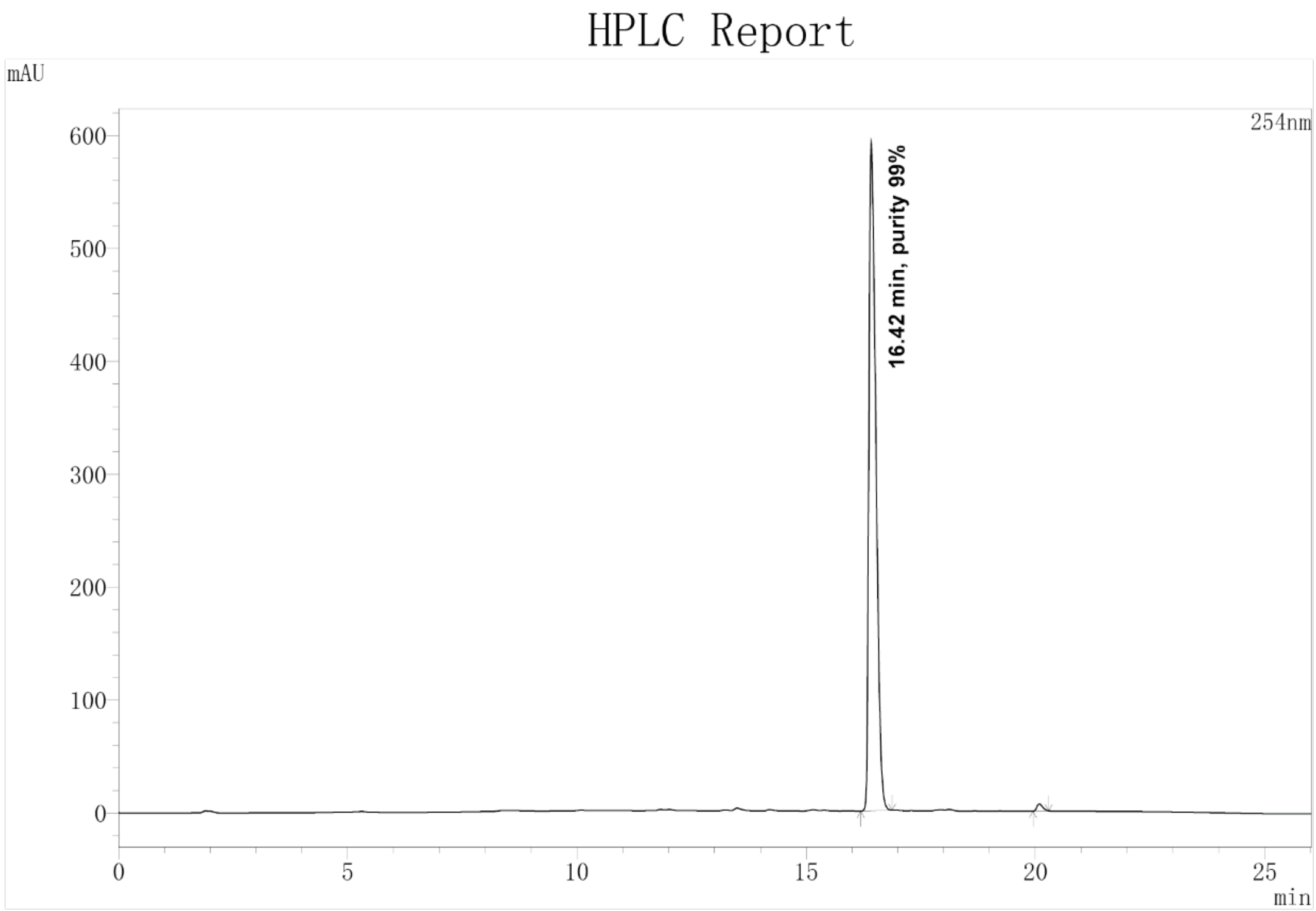

## Reference:


1 Cao, C. *et al.* Structure, function and pharmacology of human itch GPCRs. *Nature* **600**, 170-175 (2021). https://doi.org/10.1038/s41586-021-04126-6
